# Probability-of-Superiority SEM (PS-SEM)—Detecting Probability-Based Multivariate Relationships in Behavioral Research

**DOI:** 10.3389/fpsyg.2018.00883

**Published:** 2018-06-13

**Authors:** Johnson Ching-Hong Li

**Affiliations:** Department of Psychology, University of Manitoba, Winnipeg, MB, Canada

**Keywords:** probability-of-superiority, structural equation modeling, monte carlo simulation, nonlinear modeling, factor analysis

## Abstract

In behavioral research, exploring bivariate relationships between variables *X* and *Y* based on the concept of probability-of-superiority (PS) has received increasing attention. Unlike the conventional, linear-based bivariate relationship (e.g., Pearson's correlation), PS defines that *X* and *Y* can be related based on their likelihood—e.g., a student who is above mean in SAT has 63% likelihood of achieving an above-mean college GPA. Despite its increasing attention, the concept of PS is restricted to a simple bivariate scenario (*X*-*Y* pair), which hinders the development and application of PS in popular multivariate modeling such as structural equation modeling (SEM). Therefore, this study addresses an empirical-based simulation study that explores the potential of detecting PS-based relationship in SEM, called PS-SEM. The simulation results showed that the proposed PS-SEM method can detect and identify PS-based when data follow PS-based relationships, thereby providing a useful method for researchers to explore PS-based SEM in their studies. Conclusions, implications, and future directions based on the findings are also discussed.

## Introduction

Structural Equation Modeling (SEM) is one of the most widely employed statistical models in behavioral and social sciences (Bentler, 1995; Byrne, 2011). SEM aims to identify relationships among observed variables based on a conceptual model that involves two parts: measurement and structural. The measurement part examines whether or not observed variables or items (e.g., SAT) can be loaded onto the associated latent factors (i.e., reading/writing and math). The structural part examines the extent to which these latent factors are related (e.g., reading/writing and math factors regressed on test takers' intelligence). To date, SEM has been extended to complex multivariate modeling (or generalized SEM): growth curve models, robust mean modeling, multi-level modeling, and multi-level meta-analysis (e.g., Fan and Hancock, 2012; Cheung, 2015; Danner et al., 2015; Kline, 2015; Newsom, 2015; Grimm et al., 2016).

In SEM, researchers often fit a model based on the linear relationships between observed variables and latent factors (i.e., measurement part), and the relationships between latent factors (i.e., structural part) are linear. For the measurement part, an estimated factor loading between an observed variable and a latent factor (e.g., 0.80) assumes that a one unit increase in the factor is expected to produce a 0.80-unit increase in the variable (i.e., linearity assumption). For the structural part, an estimated regression slope between an exogenous latent factor and an endogenous latent factor (e.g., 0.50) also implies the linearity assumption. That is, a 1-unit increase in the exogenous factor is expected to induce a 0.50-unit increase in the endogenous factor. Indeed, the model-implied covariance matrix in SEM, a crucial matrix based on the multiplications across coefficients (e.g., factor loadings, slopes, correlations, etc.) implied by a researcher's specified conceptual model, also depends upon the linearity assumption among variables.

In addition to the linear-based SEM, researchers could also fit a nonlinear SEM, i.e., variables that are correlated based on nonlinear relationships. Curvilinear SEM is one example that allows researchers to examine any curvilinear (e.g., squared, cubic) relationships between an exogenous factor and an endogenous factor. A common approach is that researchers can add an extra slope parameter that governs the relationship between the squared scores of the exogenous factor and the raw scores of the endogenous factor for detecting curvilinear relationships (Kline, 2015).

Another type of nonlinear relationship—probability of superiority (PS)—is an important statistical concept that originates from a research scenario involving one categorical variable (e.g., treatment/control) and one continuous variable (e.g., body weight) (McGraw and Wong, 1992; Cliff, 1993; Ruscio, 2008; Li, 2016). For example, there is 70% likelihood that a randomly selected person has a lower body weight in the treatment group than a randomly selected person in the control group. There are few studies that explore PS in research scenarios involving two continuous variables. Dunlap (1994) is one of these studies and defines that PS measures the extent to which a randomly chosen score is above-mean in *X*, the paired *Y* score is also above-mean in *Y*. For example, “a father who is above average in height has a 63% likelihood of having a son of above-average height” (Dunlap, 1994, p. 510). Without exploring the full potential of PS, Dunlap only regarded PS (π_*r*_) as simple transformation from Pearson's correlation (*r*),

(1) $$\begin{array}{l} \pi_{r}=\sin^{-1}\left(r\right)/\text{pi}+.5 \end{array}$$

where *r* is Pearson's correlation, sin^−1^ is the inverse sine function and pi is constant (3.14159), such that *r* (bivariate linearity) can be translated into a metric that is more understandable and interpretable. Note that the value of 0.5 in PS corresponds to zero (or lack of) association between two variables (i.e., 50% likelihood by chance). Based on Equation (1), PS (or π_*r*_) is bound between 0 and 1 (no negative values). Recently, some studies (e.g., Brooks et al., 2014; Li, 2016) have shown that π_*r*_ is not only restricted to enhanced interpretability than *r*, but it can be extended and developed as a new modeling framework that can detect and identify PS-based bivariate relationships. In light of the potential of PS, a primary purpose of this study is to propose and develop the algorithm that can detect PS-based multivariate relationships in SEM, a popular statistical modeling in behavioral research.

The paper is divided into 4 sections. In first section, I review the background of PS-based bivariate relationships. In second section, I discuss the present development of the PS algorithms for the SEM framework, namely PS-SEM. In third section, I describe the design and methodology of the Monte Carlo experiment that compare and evaluate the performance of the estimates based on the conventional linear-based SEM and PS-SEM. In fourth section, conclusions and implications of the findings are discussed.

## Background of PS-based bivariate relationship

### Scenario 1: one categorical variable and one continuous variable

The statistical concept of PS can be traced back in the 1970s, when Wolfe and Hogg (1971) first discussed the potential of PS in behavioral research. McGraw and Wong (1992) formalized an algorithm that can measure and quantify PS in an independent-samples *t-*test data scenario. Let {$X_{i}\sim N\left(\mu_{i},\sigma_{i}^{2}\right)$; *i* = 1, 2} be jointly normally and independently distributed random variables that represent the responses to two conditions. PS quantifies a statistic that reflects PS, i.e., *P*(*X*_1_ > *X*_2_). In equation,

(2) $$\pi =\Phi \left[\left({\bar{X}}_{1}-{\bar{X}}_{2}\right)/\sqrt{s_{1}^{12}+s_{2}^{22}}\right],$$

where $\left({\bar{X}}_{1}-{\bar{X}}_{2}\right)$ is the mean difference between two groups, $s_{i}^{2}$ is the variance for group *i* = 1, 2, and Φ is the standard normal distribution function. Stated in words, PS converts an effect into a probability estimate, which examines whether a score sampled at random from distribution 1 is larger than a score sampled at random from distribution 2. A value of 0.5 indicates equivalence between the two distributions, and a value of 1 implies perfect superiority of one distribution over another. This PS estimate and its derivatives are further explained and developed in behavioral, medical, education, and social sciences in general (e.g., Ruscio, 2008; Li, 2016).

### Scenario 2: two continuous variables

It is common among behavioral researchers to evaluate bivariate relationship between two continuous variables. Pearson's correlation *r* is commonly used to quantify the association between two continuous variables. Dunlap (1994) developed Equation (1) so that *r* can be translated to PS. For example, when *r* = 0.40, this value can be converted to 0.631 through Equation (1), meaning that there is 63.1% likelihood that a father who is above-mean in height will also have a son who is also above-mean in height. People are often more familiar and understand easier with *63.1% likelihood* than *16% of variance explained* (or 16% of variance of sons' height is accounted for by his fathers' height) in this case. The improved interpretability of the PS estimates is supported in real-world research. For example, Brooks et al. (2014) conducted two experiments that recruited a sample of undergraduate students in psychology to rate their level of understandability, usefulness, and effectiveness about statistical information, which was presented as (a) proportion of variance explained (or coefficient of determination; *r*^2^), (b) probability-based common-language effect size (CLES), and (c) tabular binomial effect size display (BESD). Participants perceived both the CLES and BESD as significantly more understandable and useful than the conventional *r* and *r*^2^.

Despite the potential of π_*r*_, Dunlap (1994) stated that the two variables should be continuously and normally distributed, and π_*r*_ can only be applied in bivariate relationship involving two variables, which lead to the motivation for extending and developing the PS-based multivariate algorithm in this study.

## Development of the PS-based algorithms for the SEM framework (PS-SEM)

### Bivariate PS

Mathematically speaking, the concept of PS is beyond enhanced understanding and interpretation stated in Dunlap (1994). Rather, PS can be used to quantify PS-based bivariate relationship that cannot be detected from *r*. Or, stated differently, when the assumption of bivariate continuous normality is met, *r* is the maximum-likelihood estimator for the linear association between *X* and *Y*, when they are linearly related and bivariate normal,

(3) $$\begin{array}{l} r=\sum_{i=1}^{n}\left(x_{i}-\bar{x}\right)\cdot \left(y_{i}-\bar{y}\right)/\sqrt{\sum_{i=1}^{n}{\left(x_{i}-\bar{x}\right)}^{2}\cdot \sum_{i=1}^{n}{\left(y_{i}-\bar{y}\right)}^{2}}, \end{array}$$

where $\bar{x}=\overset{n}{\underset{i=1}{\sum }}x_{i}/n$ is the mean of scores in *X*, and $\bar{y}=\overset{n}{\underset{i=1}{\sum }}y_{i}/n$ is the mean of scores in *Y*. Possible values of *r* range from −1 to +1 (i.e., perfect-negative to perfect-positive linear correlation). Given *r* in (3), it can be converted to PS through Equation (1).

As an extension, some studies (e.g., Blomqvist, 1950; Wolfe and Hogg, 1971; Li, 2016) have already demonstrated that *X* and *Y* can be associated based on a level of PS, and this association does not depend upon the assumption of bivariate normality and linearity in *r*. Assuming that *x*_*i*_ follow a probability distribution (e.g., normal, lognormal, uniform, etc.), there exists a marginal probability distribution for *Y*_*i*_ that is generated from the following function (Blomqvist, 1950, Equation 2),

(4) $$\begin{array}{l} Y_{i}\{\begin{array}{cc} \sim U\left(\mu_{Y},c\right), & \text{if }X_{i}>\mu_{X}\text{ and }\tau \leq \gamma ,\\ \sim U\left(\mu_{Y},c\right), & \text{if }X_{i}<\mu_{X}\text{ and }\tau >\gamma ,\\ \sim U\left(-c,\mu_{Y}\right), & \text{if }X_{i}<\mu_{X}\text{ and }\tau \leq \gamma ,\\ \sim U\left(-c,\mu_{Y}\right), & \text{if }X_{i}>\mu_{X}\text{ and }\tau >\gamma ,\\ =\mu_{Y}, & \text{if }X_{i}=\mu_{X}, \end{array} \end{array}$$

where *c* is the limit in a uniform distribution, μ_*X*_ is the population mean of *X*, μ_*Y*_ is the population mean of *Y*, τ~*U*(0, 1) follows a uniform distribution with min = 0 and max = 1, and γ is the population PS that relates *X* and *Y*. Given (4), the level of probability-of-superiority between *X* and *Y* can be mathematically derived and presented as π_*p*_ (Dunlap, 1994; Li, 2016),

(5) $$\pi_{p}=\left(\sum_{i=1}^{n}\#\left[\text{sign}\left(x_{i}-\bar{x}\right)\cdot \text{sign}\left(y_{i}-\bar{y}\right)>0\right]+0.5\#\left[\text{sign}\left(x_{i}-\bar{x}\right)=\text{sign }\left(y_{i}-\bar{y}\right)=0\right]\right)/n,$$

where *n* is the number of paired *x*-*y* observations, # is the count function that counts the number of times $sign\left(x_{i}-\bar{x}\right)\cdot sign\left(y_{i}-\bar{y}\right)>0$ (or = 0), *x*_*i*_ and *y*_*i*_ are the scores from a *X*-*Y* pair in a sample, $\bar{x}$ is the sample mean of *X*, and $\bar{y}$ is the sample mean of *Y*. Conceptually, the function of $\#\left[sign\left(x_{i}-\bar{x}\right)\cdot sign\left(y_{i}-\bar{y}\right)>0\right]$ counts the number of times when an *X* score is above (or below) the mean of *X* its paired *Y* score is also above (or below) the mean of *Y*. If both the *X* and *Y* scores are identical to their corresponding means {i.e., $0.5\#\left[sign\left(x_{i}-\bar{x}\right)=sign\left(y_{i}-\bar{y}\right)=0\right]$}, then a count of 0.50 is used.

**Figure 1:**
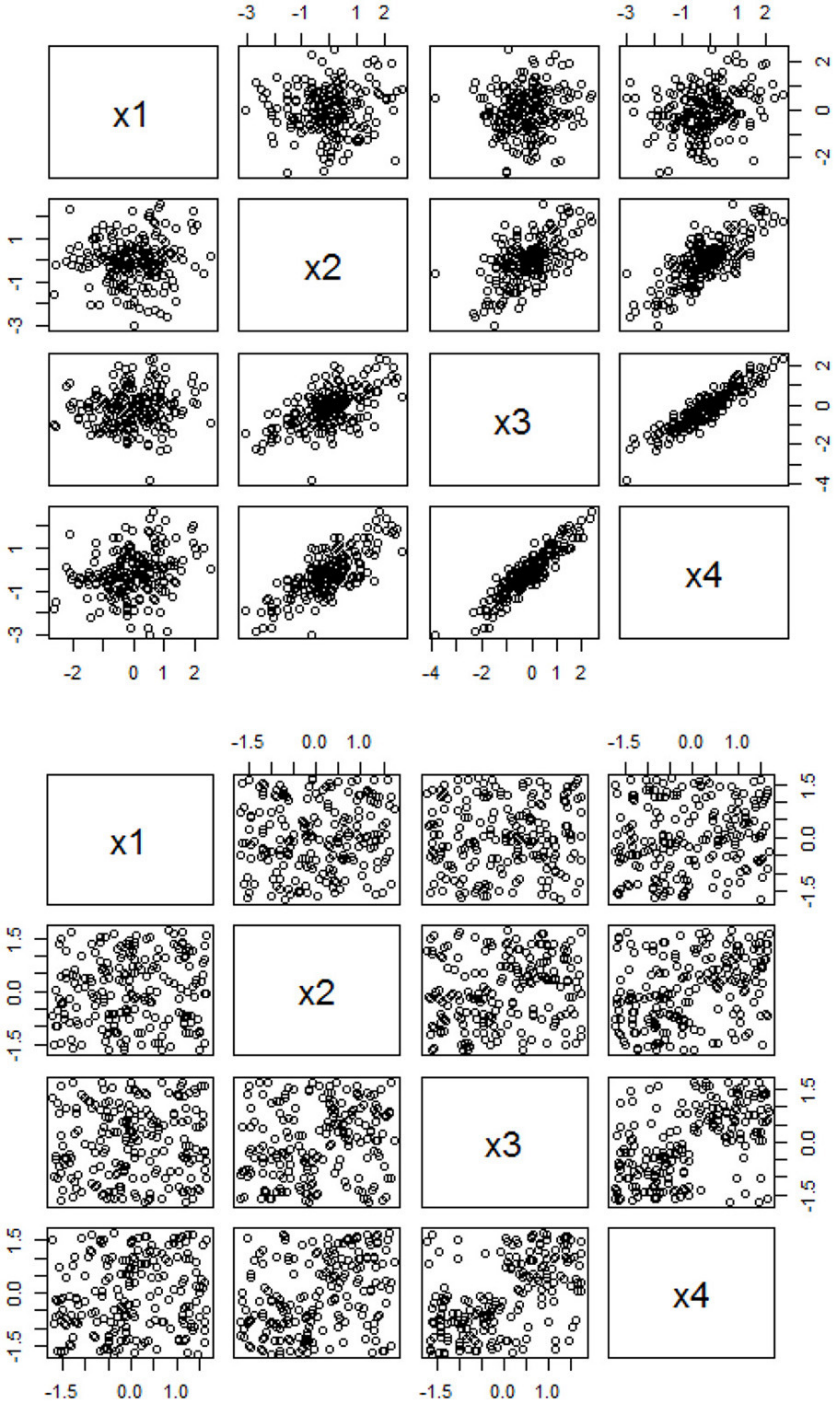
Scatterplots based on the linear-based and PS-based data. The top panel is based on the linear-based correlations among four variables with a correlation matrix of $\left[\begin{array}{cccc} 1 & .05 & .1 & .3\\ .05 & 1 & .5 & .7\\ .1 & .5 & 1 & .9\\ .3 & .7 & .9 & 1 \end{array}\right]$, and the bottom panel is based on the PS-based associations among four variables with a *r*-to-PS transformed matrix of $\left[\begin{array}{cccc} 1 & .516 & .532 & .597\\ .516 & 1 & .667 & .747\\ .532 & .667 & 1 & .856\\ .597 & .747 & .856 & 1 \end{array}\right]$, with a sample size of 200, respectively.

Figure 1 clearly shows the visual differences for bivariate relationship based on linearity and PS. The top panel of Figure 1 shows the scatterplots for *X*-*Y* that are generated from a multivariate normal distribution with a mean vector of 0 and a correlation matrix of $\left[\begin{array}{cccc} 1 & .05 & .1 & .3\\ .05 & 1 & .5 & .7\\ .1 & .5 & 1 & .9\\ .3 & .7 & .9 & 1 \end{array}\right]$, and the bottom panel shows the scatterplots for *X*-*Y* that are generated from the same mean vector but with a *r*-to-PS converted matrix of $\left[\begin{array}{cccc} 1 & .516 & .532 & .597\\ .516 & 1 & .667 & .747\\ .532 & .667 & 1 & .856\\ .597 & .747 & .856 & 1 \end{array}\right]$ based on Equation (1) and through the data generation function in Equation (4). It is likely that most researchers may believe that there are no clear or obvious patterns of relationships in the bottom panel (PS-assumed relationships), when they only use conventional *r* as an estimator for understanding relationships among variables.

### Using multivariate PS as an input covariance matrix in SEM

In behavioral research, examination of multivariate relationships in SEM has become the state-of-the-art practice in many research scenarios. SEM is a multivariate and highly flexible model that incorporates the degree and extent to which the observed variables can be loaded onto their corresponding latent variables (i.e., measurement part), and examine how the latent variables are related to one another (i.e., structural part). Statistically speaking, a researcher aims to conduct a SEM analysis such that the observed covariance matrix, **S**, is as close as to the model-implied covariance matrix, **Σ**, which is implied from the researcher's specified theory. **Σ** cannot be directly observed from a dataset. Rather, researchers should input their sample observed covariance matrix **S** among all the observed indicators in their dataset and specify their theory or causal model in the SEM framework. Next, researchers can select and use an estimator (e.g., maximum likelihood) with a statistical package [e.g., lavaan (Rosseel, 2012) in R Studio Team (2017)] that estimates the coefficients in the model-implied covariance matrix **Σ** such that the difference between **S** and **Σ** can be minimized. In this study, I propose the use of PS-based covariance elements in **S**, with the diagonal elements equal to the variances of the observed variables [*Var*(*y*_*p*_)], and the off-diagonal elements are substituted by $\pi_{pp}\cdot \sqrt{Var\left(y_{p}\right)\cdot Var\left(y_{p}\right)}$, such that

(6) $$S_{PS}=\left[\begin{array}{llll} Var(y_{1}) & \pi_{12}\cdot \sqrt{Var(y_{1})\cdot Var(y_{2})} & \cdots & \pi_{1p}\cdot \sqrt{Var(y_{1})\cdot Var(y_{p})}\\ Var(y_{2}) & \;Var(y_{2}) & \cdots & \pi_{2p}\cdot \sqrt{Var(y_{2})\cdot Var(y_{p})}\\ \vdots & \;\vdots \; & & \;\vdots \;\\ Var(y_{p}) & \pi_{p2}\cdot \sqrt{Var(y_{p})\cdot Var(y_{2})} & \cdots \; & Var(y_{p})\; \end{array}\right],$$

where π_*pp*_ for any paired variables in the covariance matrix can be estimated and obtained in (5). There are three common estimators that are commonly used and can be considered in this study.

#### Maximum likelihood (ML)

ML is the conventional approach to estimating fit and coefficients in SEM, and it was developed based on the assumption that the variables are multivariate normal. ML uses derivatives to minimize a fit function (*T*_*ML*_),

(7) $$\begin{array}{l} T_{ML}=-2\left\{-0.5\left[tr\left(\text{S}\Sigma^{-1}\right)+\log|\Sigma |-\log|\text{S}|-\left(p+q\right)\right]\right\}, \end{array}$$

where *T*_*ML*_ follows a χ^2^ distribution with degrees of freedom equals *df* = 0.5[(*p*+*q*)(*p*+*q*+1)]−*t*, where *t* is the number of estimated coefficients in SEM, and (*p* + *q*) is the number of observed indicators (*p*: endogenous, *q*: exogenous; Hayduk, 1987).

#### Weighted least squares (WLS) and unweighted least squares (ULS)

According to Olsson et al. (2000), both WLS and ULS are asymptotic free distribution functions that minimize the difference between **S** and **Σ**, and they not depend upon multivariate normality. Browne (1984), and Jöreskog and Sörbom (1982) showed that the ML, WLS, and ULS can be presented as a generalized form in which their difference depends upon the choice of a weight matrix that adjusts for any skewness or kurtosis in observed data, i.e.,

(8) $$\begin{array}{l} T_{ML}={\left(s-\;\sigma \right)}^{\prime }{W_{ML}}^{-1}\left(s-\;\sigma \right),\\ T_{WLS}={\left(s-\;\sigma \right)}^{\prime }{W_{WLS}}^{-1}\left(s-\;\sigma \right),\\ T_{ULS}={\left(s-\;\sigma \right)}^{\prime }{W_{ULS}}^{-1}\left(s-\;\sigma \right), \end{array}$$

where $\text{s}={\left({\text{s}}_{11},{\text{s}}_{21},{\text{s}}_{21},\ldots ,{\text{s}}_{\text{p}\text{p}}\right)}^{\prime }$ refer to the elements in S, $\sigma ={\left(\sigma_{11},\sigma_{21},\sigma_{21},\ldots ,\sigma_{\text{p}\text{p}}\right)}^{\prime }$ refer to the elements in **Σ**, **W**_**ML**_, **W**_**WLS**_, or **W**_**ULS**_ is a [*p*(*p*+1)]/2 by [*p*(*p*+1)]/2 weight matrix unique to ML, WLS, and ULS, respectively. Comparatively speaking, according to Olsson et al. (2000), the weight matrix for ML can be expressed as ${{\text{W}}_{\text{M}\text{L}}}^{-1}={\text{K}}_{\text{p}}^{-1}\left({\text{S}}^{-1}⊗{\text{S}}^{-1}\right){\text{K}}_{\text{p}}^{-1}$, where ${\text{K}}_{\text{p}}^{-1}$ is formally defined in (Browne, 1974). On the other hand, the elements in ${{\text{W}}_{\text{W}L\text{S}}}^{-1}$are only functions of second- and fourth-order moments of the observed variables in order to adjust for skewness and kurtosis, and **W**_**ULS**_ is the identity matrix in ULS.

### Goodness-of-fit indices in PS-SEM

In practice, researchers have to check for the accuracy and goodness-of-fit indices of **Σ**, their specified causal model, because a mis-specified model often inappropriately links the true casual relationships among observed indicators. It is rare that researchers may question about the accuracy of their inputted observed covariance matrix **S**. Specifically, if any possible pairwise observed indicators in **S** are associated based on PS instead of the bivariate linearity assumption in conventional Pearson's correlation *r*, then the inputted coefficients in **S** are inaccurate, which cannot reflect the true associations among the observed indicators (i.e., Equations 4 and 5). Hence, this study proposes the use of PS coefficients in (6) as the input elements in the observed covariance matrix **S** for the SEM analysis. This approach could explore and open a new era that conventional, linearly based SEM cannot detect the PS-based multivariate relationships.

In conventional linear-based SEM, researchers are interested in examining the test function values (i.e., *T*_*ML*_, *T*_*WLS*_, and *T*_*ULS*_) with a smaller value means a smaller discrepancy (or better fit) between **S** and **Σ**. *T*_*ML*_ and *T*_*WLS*_ values are expected to follow a χ^2^ distribution with *df* = 0.5[(*p*+*q*)(*p*+*q*+1)]−*t*, whereas *T*_*ULS*_ produces an unscaled value that do not follow a χ^2^ distribution. In addition, researchers often report and interpret many different types of goodness-of-fit indices—e.g., CFI, RMSEA—as supplementary to the χ^2^ test (Jackson et al., 2009). In this study, I propose the use of PS-based observed covariance matrix (**S**_**PS**_ in Equation 6) and plugged into the three estimators. Given that the original fit functions (*T*_*ML*_, *T*_*WLS*_, and *T*_*ULS*_) were developed based on the conventional linear-based bivariate correlations between the observed variables, the fit functions based on **S**_**PS**_ may compromise and may not be comparable with the linear-based estimates coming from *T*_*ML*_, *T*_*WLS*_, and *T*_*ULS*_.

## Monte carlo simulation

A Monte Carlo simulation study is regarded as a computer-simulated experiment that aims to assess the accuracy or robustness of a statistical method across a number of replicated samples. The purpose of the current simulation is to examine whether the conventional linear-based estimators and the proposed PS-based estimators could produce accurate or robust parameter estimates (e.g., factor loadings, factor correlations, etc.) and goodness-of-fit indices (e.g., CFI, RMSEA, etc.) in SEM/CFA, when variables are related based on linear or PS relationships. The goal of this simulation is to offer empirical evidence and guidelines for researchers to choose an appropriate estimation method for obtaining these parameter estimates and fit indices, when their data are linear- or PS-related.

### Design

A key feature of the simulation study is to mimic a realistic research scenario that is often found in behavioral research. According to DiStefano and Hess's (2005) review of 100 empirical SEM (or confirmatory factor analysis; CFA) studies, they showed clear guidelines about the common factor structure—i.e., number of factors, factor loadings, and factor correlations—in behavioral research. Specifically, they found that a typical SEM/CFA model in behavioral research consists of four correlated factors with a median value of 0.30, and each factor consists of five items or indicators with a median factor loading of 0.70. Moreover, DiStefano and Morgan's (2014) Monte Carlo experiment follow these guidelines in simulating observations for the SEM/CFA model, and evaluate the performance of parameter estimates and goodness-of-fit indices based on different estimators (e.g., ML, robust ML) by different levels of sample sizes (i.e., 400, 800, 1200, 1600) and scales of measurement (i.e., 2-, 3-, 5-, 7-point). This also provides guidelines for the design of the current Monte Carlo experiment, meaning that the factor correlations are fixed at 0.30, and factor loadings are fixed at 0.70. The scores are generated from the conventional linear-based correlation matrix and the PS-based matrix, and their parameter estimates are compared with the true population values.

**1. Sample size (4 levels)**. Three levels are examined, including 400, 800, 1,200, and 1,600, which follow a similar design in DiStefano and Morgan (2014).

**2. Scale of measurement (10 levels)**. Following DiStefano and Morgan (2014), this study evaluates five types of measurement: continuous normal, 2-point, 3-point, 5-point, and 7-point scales for data that follow the conventional linear-based relationships. Moreover, this study also examines five types of PS-based types of measurement: continuous uniform, 2-point, 3-point, 5-point, and 7-point scales based on the function in (4).

Regarding data generation, without loss of generality, observations were first generated from a multivariate normal distribution, *N*~(**μ**, **Σ**) (or the ordinal form, 2-, 3-, 5-, and 7-point, of multivariate normal data based on the function ordsample in RStudio), where **μ** is a 20 (i.e., number of items) by 1 vector that contains all zeros, and **Σ** is a 20 by 20 covariance/correlation matrix that presents the true population correlation among items based on the manipulated level of factor correlation and factor loading. These distributions refer to the data scenarios, in which the assumption of linearity is met.

Moreover, to convert the continuous correlation-based scores *x*_*ij*_, where *i* refers to 1,…, *n* observations, and *j* indicates 1,…,20 item, into continuous PS-based scores *z*_*ij*_, *z*_*ij*_ was generated from a uniform distribution with min = $-\sqrt{12}/2$ and max = 0 when *x*_*ij*_ is smaller than (or equal to) its item mean $\bar{x_{j}}$, and from a uniform distribution with min = 0 and max = $\sqrt{12}/2$ when *x*_*ij*_ is larger than its item mean $\bar{x_{j}}$. That is,

(9) $$z_{ij}\{\begin{array}{ll} U\sim (0,\sqrt{12}/2), & when\;x_{ij}>\bar{x_{j}}\;\\ U\sim (-\sqrt{12}/2,0), & when\;x_{ij}\leq \bar{x_{j}} \end{array}.$$

The use of $\pm \sqrt{12}/2$ is to ensure that the expected SD of the generated *z*_*ij*_ scores equals 1 without loss of generality. For generating the 2-point PS-based scores, the original *x*_*ij*_ scores were transformed to binary scores with the condition that

(10) $$\begin{array}{l} z_{ij}\{\begin{array}{cc} \;1, & \;when\;x_{ij}>\bar{x_{j}}\;\\ 0, & when\;x_{ij}\leq \bar{x_{j}} \end{array}\;. \end{array}$$

For generating the 3-, 5-, and 7-point PS-based scores, the original *x*_*ij*_ scores were converted to uniform integer distribution,

(11) $$z_{ij}\{\begin{array}{ll} \mathbb{Z}\sim (0,c), & when\;x_{ij}>\bar{x_{j}}\;\\ \mathbb{Z}\sim (c,0), & when\;x_{ij}\leq \bar{x_{j}} \end{array},$$

where *c* is the cutoff value (or medium value) of the point scale manipulated in the study, i.e., *c* = *b*/2+.01, where *b* is the number of points manipulated in a scale. The inclusion of 0.01 is to ensure that the point-scale PS-categorized scores are still uniformly and evenly distributed.

Three estimations are examined. First, the conventional maximum likelihood (ML) estimator for linear-based SEM is evaluated. Second, given that the ML estimator depends upon the standard parametric assumption (multivariate normality), the ML estimation with robust (Huber-White) standard errors (MLR), WLS, and ULS in the lavaan package are also examined. Third, the proposed PS-based covariance matrix (**S**_**PS**_) serving as an input in the ML, WLS, and ULS (i.e., PS-ML, PS-WLS, and PS-ULS) are examined.

In sum, this study produces a total design with 4 × 10 = 40 conditions. Each condition was replicated 1,000 times, producing a total of 40,000 simulated data-sets. Each data-set is used to compute the factor loadings, factor correlations, and goodness-of-fit indices (χ^2^, CFI, and RMSEA) based on PS-ML, PS-WLS, PS-ULS, ML, MLR, WLS, and ULS estimators, respectively. The code was executed in RStudio (R Studio Team, 2017), with the package MASS loaded for generating the multivariate data (Venables and Ripley, 2002) and the package lavaan (Rosseel, 2012) for running and executing a CFA/SEM analysis. The simulation code is shown in the supplementary materials.

### Evaluation criteria

Percentage bias is used to evaluate the performance of the estimated factor correlations and factor loadings, i.e., $bias=\left(\bar{\gamma }-\varphi \right)/\varphi$, where $\bar{\gamma }$ is the mean of 1,000 replicated factor correlations or loadings, and φ is the associated true value in the population level. According to Li et al. (2011), bias that is within ±0.10 (or ±10%) is considered reasonable. Moreover, the standard error (SE) of the parameter estimates (factor loadings and correlations) is important for researchers to understand and compare the sampling errors based the linear-based and PS-based estimators. Regarding the model fit, a lower χ^2^ indicates a better fit between the observed and model-implied covariance matrices. In addition, a CFI larger than .90 and a RMSEA smaller than 0.08 are often considered a reasonable fit in practice.

## Results

### Biases of parameter estimates (Figure 2)

#### Linear-based data

**Figure 2:**
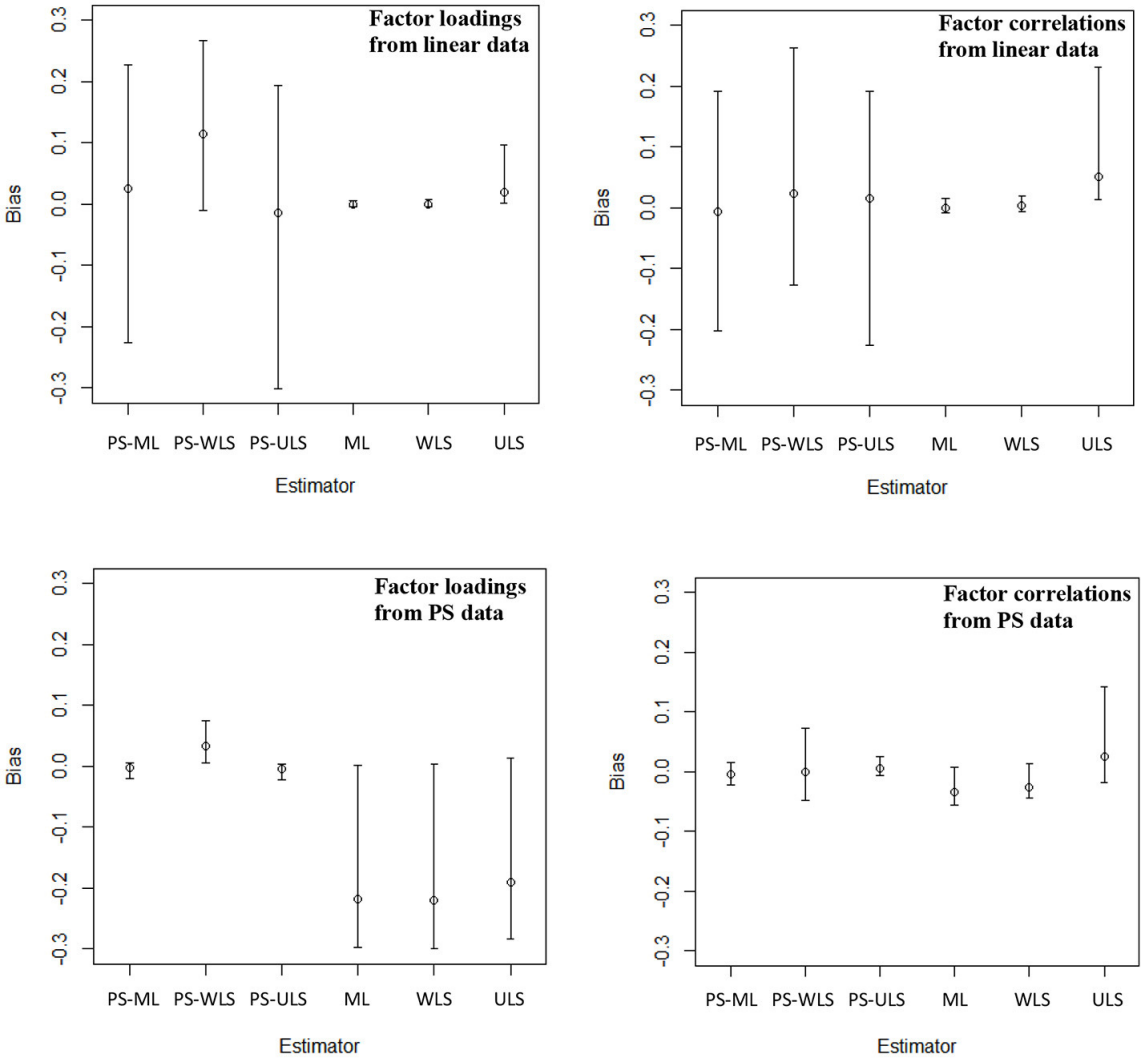
Biases of the factor loadings and factor correlations when data are linear or PS-related. The top left panel shows the mean, min, and max of the biases of 20 factor loadings across 40 conditions, and the top right panel shows the mean, min, and max of the biases of 6 factor correlations across 40 conditions, when data are linear-based. The bottom left panel shows the mean, min, and max of the biases of 20 factor loadings across 40 conditions, and the bottom right panel shows the mean, min, and max of the biases of 6 factor correlations across 40 conditions, when data are PS-based.

First, the ML and MLR estimators result in the same results. This is because the MLR estimator only adjusts for the standard errors of the estimates, and this estimator results in the same sample estimates through the likelihood maximization as in the ML estimator. Hence, the biases of the MLR are dropped in Figure 2. Second, the conventional ML, WLS, and ULS estimators produce highly accurate results as predicted, when data are linearly related. For ML, the biases range from −0.001 to 0.001 with a mean of 0.000 [i.e., range = (−0.009, 0.015), mean = 0.000]. For WLS, range = (0.002, 0.232) and mean = 0.028. For ULS, range = (−0.006, 0.020) and mean = 0.001. Third, the proposed PS-based estimators, however, produce estimates that are more biased than the conventional linear-based ML, WLS, and ULS estimators, when variables are linearly related to one another. Regarding PS-ML, the range of the biases was (−0.226, 0.228) with a mean of .017. PS-WLS produces upward biases: range = (−0.128, 0.268) and mean = 0.093. For PS-ULS, range = (−0.302, 0.193) and mean = −0.008. These results suggest that researchers should only use the conventional linear-based estimators, if they observe that their data are linearly related.

**Figure 3:**
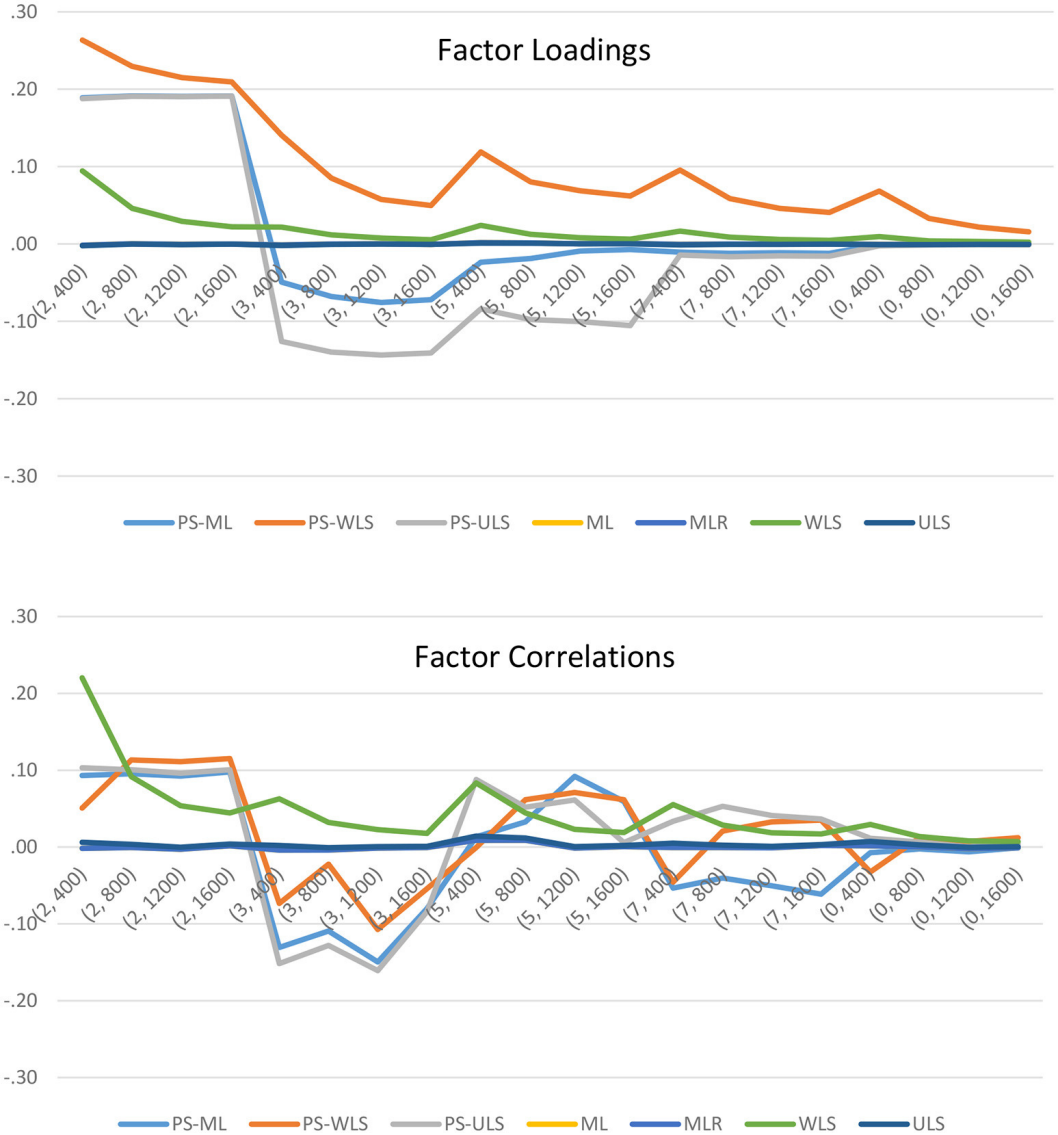
Biases of the factor loadings and factor correlations when data are linearly related. The horizontal axis shows the 20 simulation conditions, where the first number of the bracket indicates the scale of measurement (i.e., 2 = 2-point, 3 = 3-point, 5 = 5-point, 7 = 7-point, and 0 = continuous normal), and the second number refers to the sample size.

To further examine how the manipulated factors influence the variability of the biases, we can refer to the results in Figure 3. When the number of points increases from 2 to infinity (i.e., continuous normal), the biases of the three PS-based (i.e., PS-ML, PS-WLS, and PS-ULS) factor loadings and factor correlations decrease, and these biases become closer to the biases obtained through most of the conventional linear-based estimators such as ML, MLR, and ULS. Of the 3 PS-based estimators, PS-ML appears to produce more reasonable factor-loading and factor-correlation estimates, when the number of points becomes 7 or data become continuous normal. However, this estimator is still less accurate than ML, MLR, or ULS. In sum, researchers should use the conventional linear-based estimators, when data are linearly related in their sample.

#### PS-based data

When data follow PS-based relationships, the conventional estimators (ML, WLS, and ULS) produce noticeable downward-biased parameter estimates, as shown in Figure 2. For ML, the 20 factor loadings and 6 factor correlations contain biases with range = (−0.219, 0.007) and mean = −0.176. For WLS, range = (0.283, 0.142) and mean = −0.141. For ULS, range = (−0.299, 0.013), mean = −0.175. Comparatively, the proposed PS-based estimators produce more accurate estimates than ML, WLS, and ULS. For PS-ML, the biases of the 20 factor loadings and 6 factor correlations are minimal with range = (−0.022, 0.015) and mean = −0.003. For PS-WLS, range = (−0.047, 0.075) and mean = 0.025. For PS-ULS, range = (−0.023, 0.026) and mean = −0.001.

**Figure 4:**
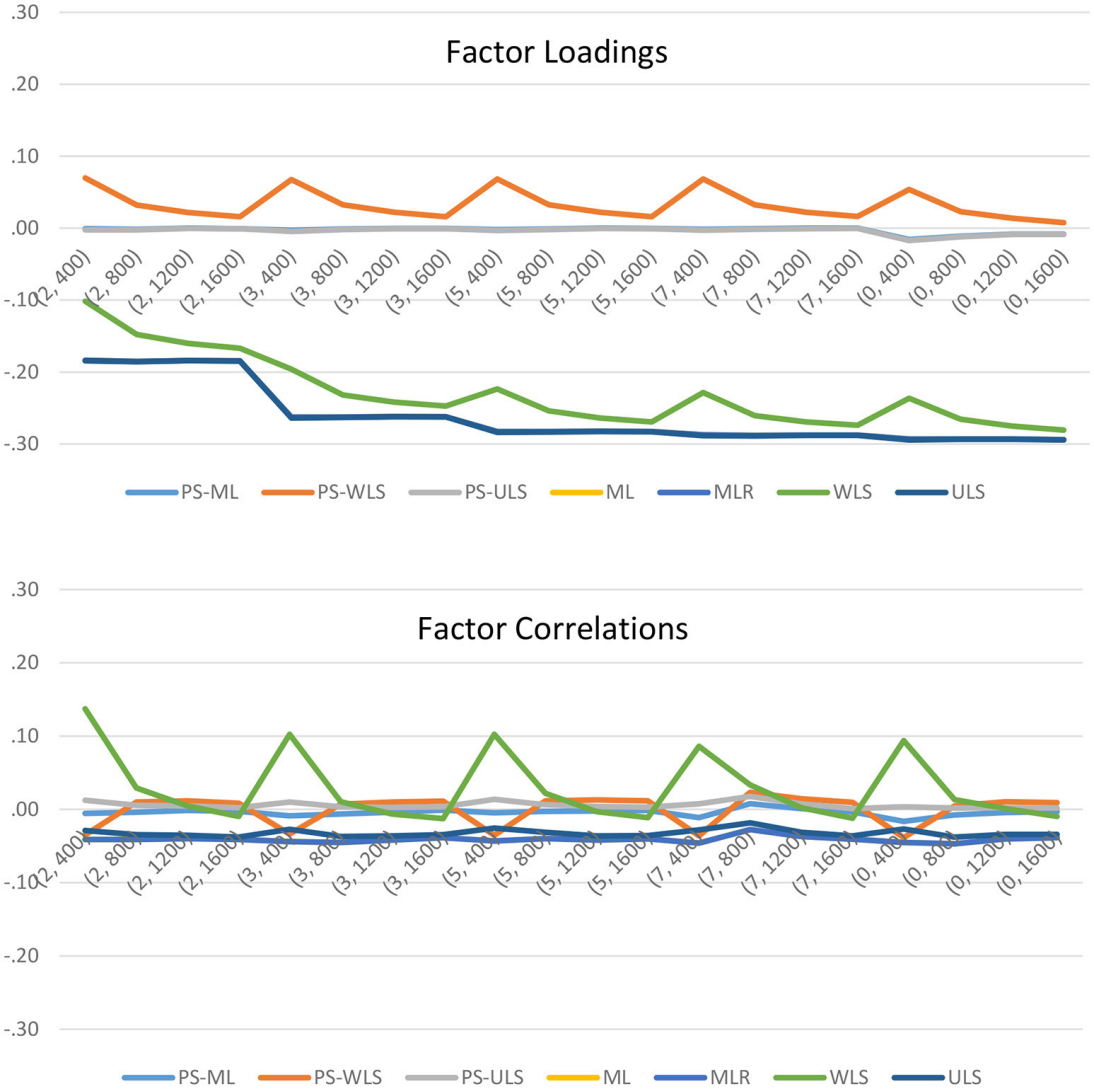
Biases of the factor loadings and factor correlations when data are PS-related. The horizontal axis shows the 20 simulation conditions, where the first number of the bracket indicates the scale of measurement (i.e., 2 = 2-point, 3 = 3-point, 5 = 5-point, 7 = 7-point, and 0 = continuous uniform), and the second number refers to the sample size.

To further examine the effects of the manipulated factors on the parameter estimates, we can refer to the results in Figure 4. When data are PS-related, both the PS-ML and PS-ULS produce accurate factor-loading and factor-correlation estimates. The remaining PS-based estimator, PS-WLS, only produce reasonable estimates when sample size increases from 400 to 1600. Comparatively, the conventional linear-based estimators, ML, MLR, WLS, and ULS, produce downward-biased factor-loading estimates, when data are PS-related. Regarding factor correlations, ML, MLR, and ULS also produce downward-biased estimates across the 20 conditions with PS-based data. WLS may result in slightly less biased factor-correlation estimates, when sample size increases from 400 to 1600, but this estimator is still less accurate than PS-ML or PS-ULS. In sum, researchers should use the PS-based estimators, when data are PS-related in their sample.

### SEs of the parameter estimates (Figure 5)

The PS-based estimators produce slightly more precise estimates than the conventional estimators, when data are linear-based. Specifically, the PS-ML estimator produces SE values with range = (0.007, 0.054) and mean = 0.020, for the factor correlations and loadings. For PS-WLS, range = (0.009, 0.058) and mean = 0.022. For PS-ULS, range = (0.005, 0.136) and mean = 0.039. For the conventional estimators, the ML estimator produces the SD values with range = (0.016, 0.056) and mean = 0.026. For MLR, range = (0.016, 0.058) and mean = 0.027. For WLS, range = (0.015, 0.042) and mean = 0.022. For ULS, range = (0.005, 0.149) and mean = 0.045.

**Figure 5:**
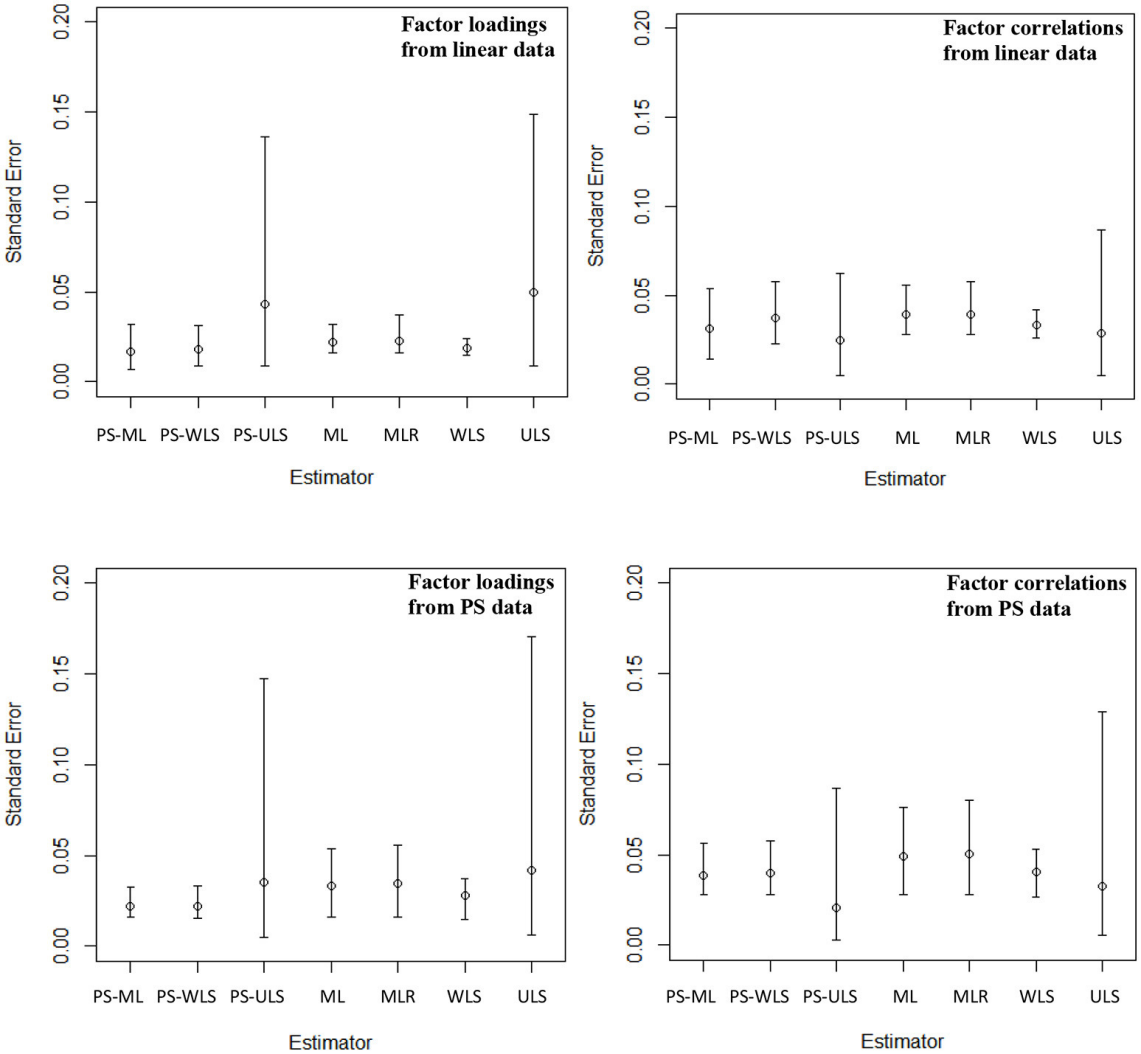
Standard errors of the factor loadings and factor correlations across 40 conditions. The top left panel shows the mean, min, and max of the standard errors of 20 factor loadings across 40 conditions, and the top right panel shows the mean, min, and max of the standard errors of 6 factor correlations across 40 conditions, when data are linear-based. The bottom left panel shows the mean, min, and max of the standard errors of 20 factor loadings across 40 conditions, and the bottom right panel shows the mean, min, and max of the standard errors of 6 factor correlations across 40 conditions, when data are PS-based.

When data follow the PS-based relationships, the PS-based estimators also produce more precise (or smaller) SE values than the conventional estimators. For PS-ML, range = (0.016, 0.056) and mean = 0.026. For PS-WLS, range = (0.016, 0.058) and mean = 0.026. For PS-ULS, range = (0.003, 0.148) and mean = 0.032. Comparatively, the SE values for the factor loadings and correlations are generally less precise based on ML with range = (0.016, 0.076) and mean = 0.037. For MLR, range = (0.016, 0.080) and mean = 0.038. For WLS, range = (0.015, 0.053) and mean = 0.031. For ULS, range = (0.005, 0.171) and mean = 0.040.

### Goodness-of-fit indices

**Figure 6:**
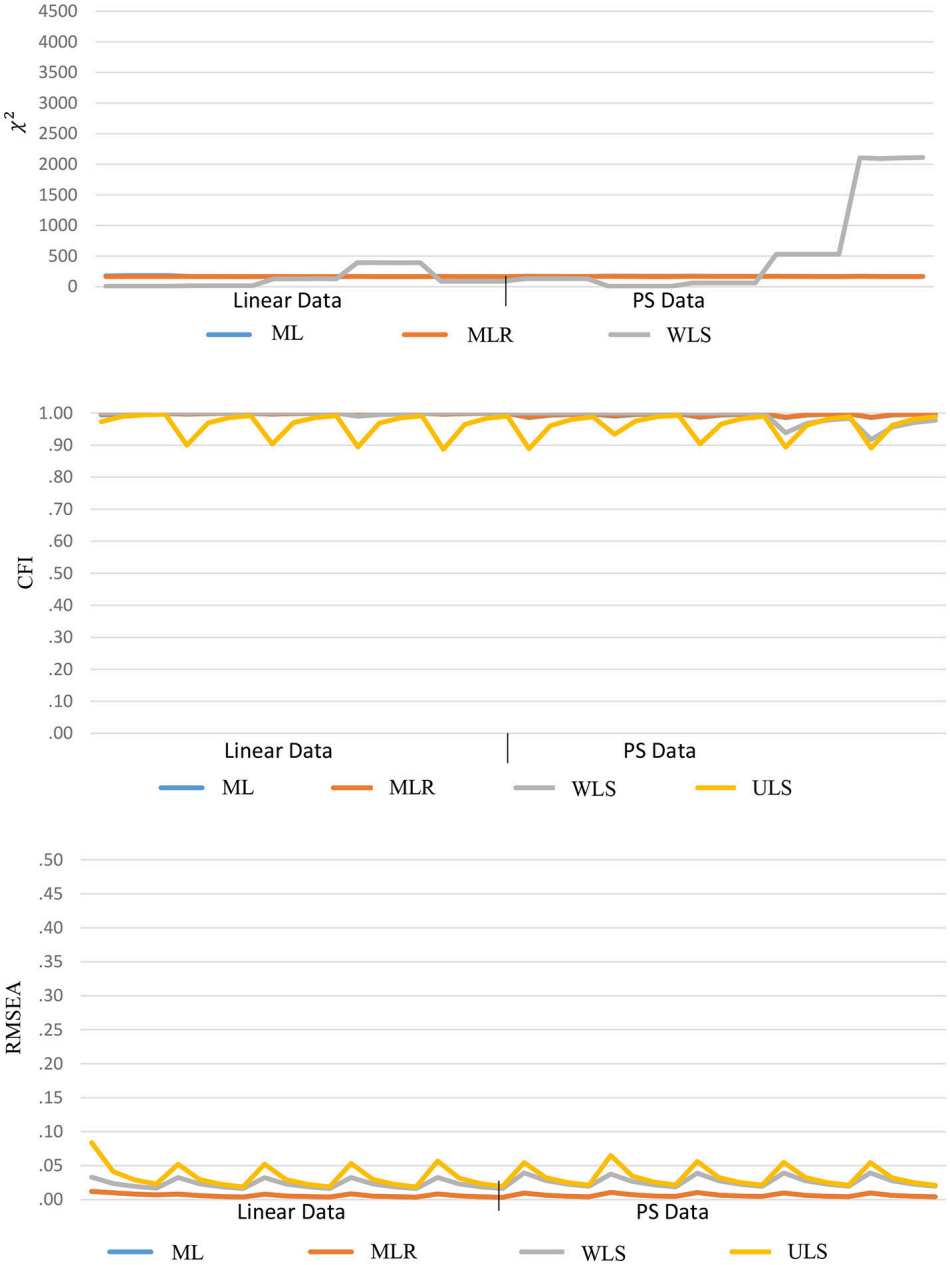
Goodness-of-fit indices based on linear estimators across 40 conditions.

Both ULS and PS-ULS produce unscaled fit-function values that are not distributed as χ^2^ values, and hence, these results are not reported in this section. The goodness-of-fit indices yielded good results based on the conventional ML, MLR, WLS, and ULS estimators, no matter whether the data are linear-based or PS-based, as shown in Figure 6. For χ^2^, the ranges are (164.51, 169.55), (163.34, 168.93) and (5.78, 2111.50), and the means are 169.55, 166.28, and 346.18, respectively, based on ML, MLR, and WLS. For CFI, the ranges are (0.986, 0.999), (0.987, 0.999), (0.918, 1) and (0.887, 0.997), and the means are 0.996, 0.996, 0.992, and 0.963, respectively, based on ML, MLR, WLS, and ULS. For RMSEA, the ranges are (0.003, 0.012), (0.003, 0.012), (0.016, 0.039) and (0.019, 0.084), and the means are 0.006, 0.006, 0.025, and 0.034, respectively, based on ML, MLR, WLS, and ULS. Note that a general pattern of good fitted results from these estimators does not mean that the results are desirable, especially when data are PS-based. This is because researchers tend to conclude that the data fits the hypothesized model well-based on the desirable fit indices, but they may underestimate the parameter estimates (i.e., factor loading and correlation; Figure 2) and misinterpret the size of the effects in the model, when data follow a PS-based distribution.

**Figure 7:**
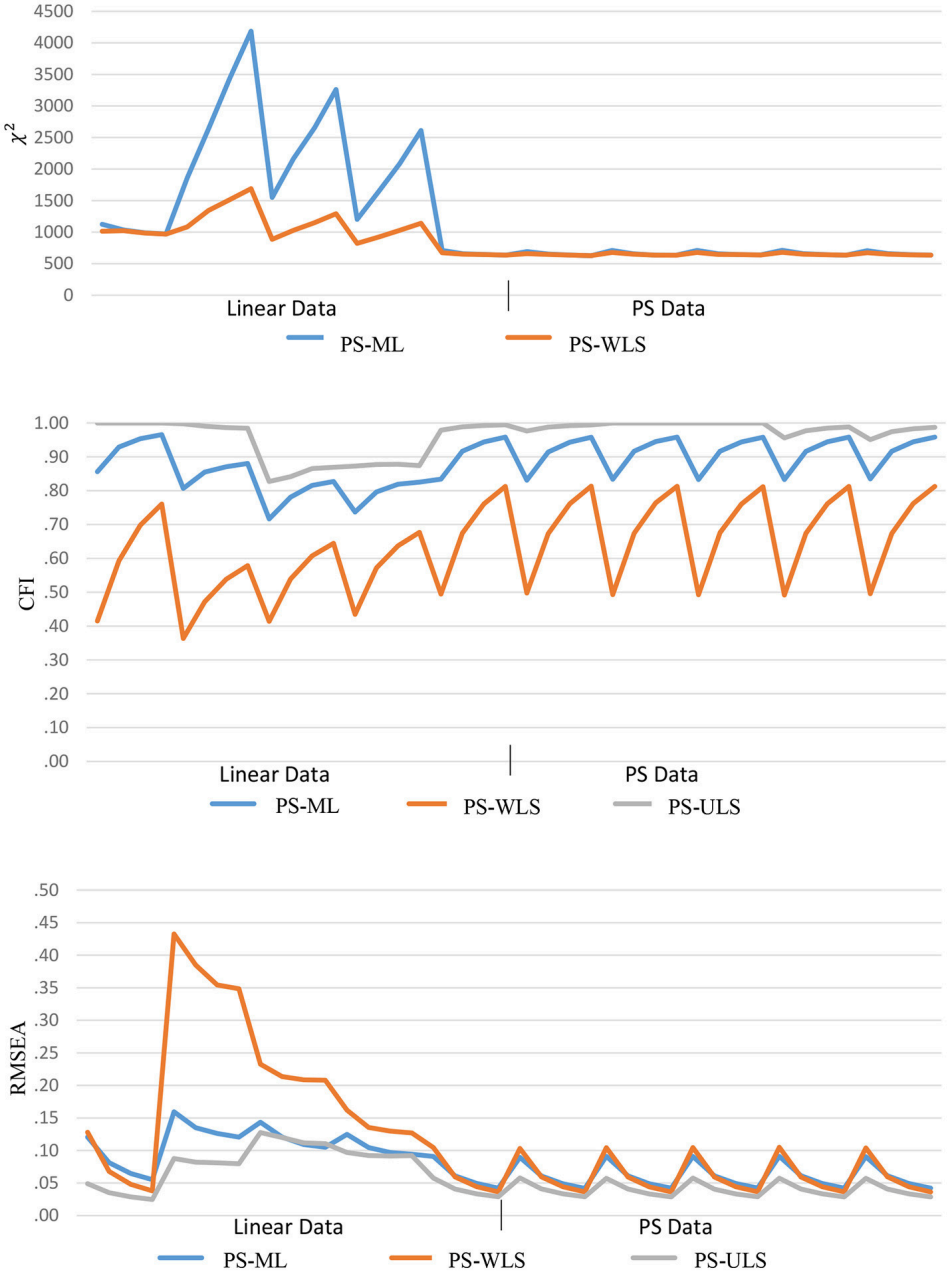
Goodness-of-fit indices based on PS estimators across 40 conditions.

For the PS-based estimators, the χ^2^, CFI, and RMSEA values are found to be less desirable when data are linear-based than PS-based, as shown in Figure 7. For χ^2^ under linear-based data, the ranges are (635.20, 4185.02) and (635.86, 1689.01), and the means are 1802.68 and 1024.49, respectively, based on PS-ML, and PS-WLS. For CFI, the ranges are (0.717, 0.966), (0.364, 0.812) and (0.827, 1), and the means are 0.854, 0.584, and 0.941, respectively, based on PS-ML, PS-WLS, and PS-ULS. For RMSEA, the ranges are (0.042, 0.159), (0.036, 0.433), and (0.025, 0.128), and the means are 0.100, 0.173, and 0.074, respectively, based on PS-ML, PS-WLS, and PS-ULS.

Comparatively, when data follow a PS-based distribution, the fit indices improve substantially. For χ^2^, the ranges are (625.13, 712.09) and (648.77, 627.15), and the means are 659.28 and 648.77, respectively, based on PS-ML and PS-WLS. For CFI, the ranges are (0.831, 0.958), (0.686, 0.492) and (0.951, 0.988), and the means are 0.913, 0.686, and 0.988, respectively, based on PS-ML, PS-WLS, and PS-ULS. For RMSEA, the ranges are (0.042, 0.091), (0.036, 0.105) and (0.029, 0.058), and the means are 0.016, 0.061, and 0.040, respectively, based on PS-ML, PS-WLS, and PS-ULS. These results show that, when data indeed follow a PS-based distribution in population, the conventional goodness-of-fit indices (χ^2^, CFI, RMSEA) provide a decent diagnostic tool for researchers to examine whether a hypothesized model fits to the observed data, and these indices perform more desirably in the PS-based data than the linear-based data.

## Working example

Appendix A shows the step-by-step procedures of how to obtain the results from the PS-based SEM analyses. A real-world data-set can be found on the online website (Raw Data from Online Personality Tests, 2018) that includes the raw scores of 973 valid respondents, who responded to the four factors or domains [i.e., assertiveness (AS), social confidence (SC), dominance (DO), and adventurousness (AD)] of the 40 experimental, 5-point Likert-scale items for the DISC Personality Test (2018). Details of the items and their association with the corresponding domains are documented in the associated codebook file through the online link.

**Figure 8:**
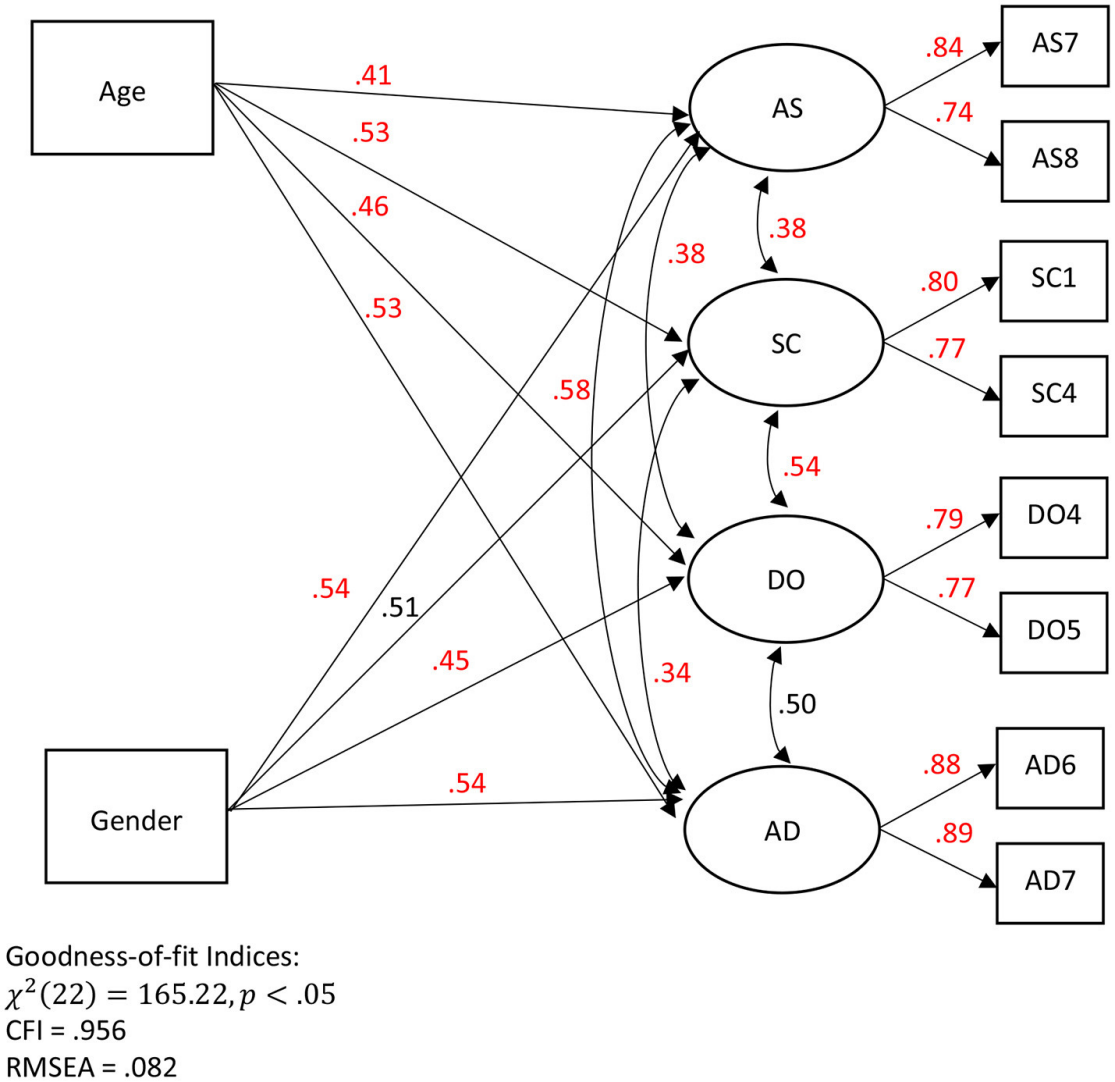
An interpreted SEM with the PS-ML estimates. AS refers to assertiveness, SC refers to social confidence, DO refers to dominance, and AD refers to adventurousness. PS-based estimates with *p* < 0.01 are presented in red.

For demonstration purposes, I am interested in examining whether age and gender could significantly predict factors on AS, SC, DO, and AD. The original model consists of a 4-correlated factor structure, and each factor is loaded onto 10 items, respectively (Raw Data from Online Personality Tests, 2018). However, according to previous studies (e.g., Hayduk, 1987), it is not necessary for researchers to include all the items in a SEM because this would increase the number of estimated parameters (i.e., factor loadings) that are redundant in measuring the associated factors, especially when the focus is on examining the regression effects in the structural part. Rather, researchers can select 2–4 items that possess the largest associations with the associated factors. This practice can reduce the number of unnecessary parameters and simplify a SEM for a higher chance of convergence with solution. In light of this, I decided to simplify the model through a series of preliminary analyses based on linear-based correlations, PS-based correlations, and exploratory factor analyses (with PS-ML and ML estimators), with the goal to select 2 items per factor. In addition, I am interested in whether age and gender (1 = male, 2–female) could significantly predict the factors of AS, SC, DO, and AD, respectively. Eventually, I have found a final interpreted model (Figure 8) after a series of model re-specification processes, which is regarded as an acceptable practice according to the APA Task Force (Appelbaum et al., 2018).

**Figure 9:**
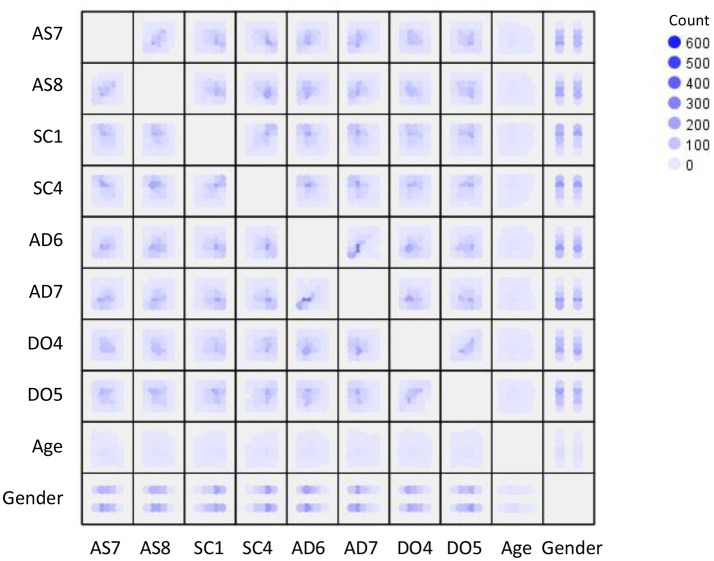
Scatterplots with heat density for the variables in the DISC Personality Test. AS refers to assertiveness, SC refers to social confidence, DO refers to dominance, AD refers to adventurousness.

Given this interpreted model, I had to decide whether the variables involving in the model are linear- or PS-related so that I can choose a linear- or PS-based estimator for the SEM analysis. As shown in Figure 9, most of the possible bivariate relationships in the hypothesized model do not show a clear pattern of linear relationship. Rather, these bivariate relationships appear to follow a PS-based relationship similar to the pattern shown in the bottom panel of Figure 1. Hence, my decision was to estimate the parameters for the hypothesized model based on the PS-ML estimator.

To obtain the PS-based results, one can use the pr function in Appendix A, pr(data, cor = FALSE), where “data” refers to the dataset imported to R, and “cor = FALSE” means that a PS-adjusted covariance matrix is outputted as default, unless cor is stated as true to obtain a PS-adjusted correlation matrix. Given this function, one can test a SEM/CFA model based on the conventional cfa function in lavaan, i.e., cfa(HS.model, estimator = “ML,” sample.cov = pr(data), sample.nobs = n, std.lv = TRUE), in order to obtain the PS-ML results, in Appendix A.

As shown in Figure 8, the goodness-of-fit indices are reasonable, especially for the purposes of demonstrating the potential of the proposed PS-ML estimator in an open-access data-set. χ^2^ test produces a significant result, χ^2^(22) = 165.22, *p* < .05, meaning that the observed covariance matrix may not fit to the hypothesized model. However, it is common that the χ^2^ test is often over-sensitive with a large sample size (e.g., *n* > 400; Kenny, 2015) in reaching this conclusion, and hence, other fit indices are also evaluated. In this case, CFI = 0.954 is larger than the criterion of 0.95, and RMSEA = 0.082 is on the edge of meeting the criterion for a reasonable fit (<0.08).

Regarding the parameter estimates, first, the PS-based factor loadings range from 0.74 to 0.89 with a mean of 0.81. For example, when someone's score on the factor (AS: assertiveness) is above the mean of the AS factor scores, there is 84% likelihood that this person's observed score on AS7 (i.e., question #7 of AS) is above the mean of the observed AS7 scores. Second, the PS-based regression estimates range from 0.41 to 0.54 with a mean of 0.50. Of the 8 regression estimates, 7 are statistically significant. Specifically, age is a significant predictor of AS, SC, DO, and AD, respectively. Taking one example for interpretation, when someone's age is above the mean age of all other participants (35.55 years old), there is 41% (or 59%) likelihood that this person's AS factor score is above (or below) the mean of the AS factor scores. Gender is a significant predictor of AS, DO, and AD, respectively. Given that gender is a categorical variable, when someone's gender score is above the mean of all other gender scores, this is equivalent to saying that when gender changes from (1 male) to 2 (female) given a relatively balanced gender ratio. Taking one example for interpretation, there is 54% likelihood that female participants score higher on AS than male participants. Third, the PS-based factor correlations range from 0.34 to 0.58 with a mean of 0.46. Taking one example for interpretation, when someone's AS factor score is above the mean of the AS factor scores, there is 38% (or 62%) likelihood that this person's SC factor score is above (or below) the mean of the SC factor scores. It is noteworthy that the interpretations of the PS-based coefficients are different from the conventional regression (slope) coefficients or correlation coefficients (*r*) in the linear model. For the case of slope and correlation coefficients, the value of 0 indicates zero (or lack of) association between variables. For the case of PS-based coefficients, the value of 0.5 corresponds to zero (or lack of) association between variables (i.e., 50% likelihood by chance).

## Conclusion and discussion

In light of the increasing attention of the PS-based bivariate relationships, this study aims to explore the potential of applying PS-based relationship in the framework of CFA/SEM, a modern, widely employed multivariate latent variable modeling in behavioral research. The proposed PS-based method (e.g., PS-ML) provides a good statistical tool for researchers to estimate the parameters (e.g., factor loading, factor correlation) in CFA/SEM, especially when they are interested in understanding how the variables are related to one another based on the concept of PS in addition to linear-based SEM.

The simulation results show that researchers can use the proposed PS-based estimators (e.g., PS-ML) to obtain good parameter estimates (e.g., factor loadings, factor correlations, etc.), when data are PS-related in their sample. If researchers use the conventional estimators (e.g., ML, MLR, WLS, or ULS) for estimating the parameters in their hypothesized SEM with the PS-based data, then they will obtain downward-biased estimates (i.e., factor loadings). On the other hand, when data are linearly related, researchers should stick with and use the conventional linear-based estimators in obtaining the parameter estimates, given that the PS estimators tend to produce estimates with more variability (i.e., either under-estimation or over-estimation across the 20 simulation conditions). In practice, researchers can first visualize a scatterplot for their variables, and decide whether they would choose a linear- or PS-based estimator for their hypothesized model. When data are linearly related based on the scatterplot, a conventional linear-based estimator should be the most appropriate. On the other hand, when data are PS-related, researchers should choose a PS-based estimator (e.g., PS-ML), and this would result in less biased parameter estimates. This study tests and develops different types of PS-based estimators so that researchers can test their hypothesized model with estimators that can estimate PS-based relationships in addition to the conventional linear-based relationships, when they find that their sample data appear to be PS-related. It is noteworthy that when a scatterplot does not show a linear pattern, there could be two possibilities: (1) the data could have the PS-based relations, or (2) there is no relation at all. Hence, researchers should check whether their data have the PS-based relations and use a PS-SEM estimator, when the scatterplot does not show a linear pattern.

This study offers a free, easy-to-execute code in R or RStudio so that researchers can conveniently explore whether or not their data adhere to a PS-based SEM than the conventional linear-based SEM, especially when they cannot find any clear linear pattern of relationships based on correlational analysis. In this case, applied researchers can visualize the data points in a scatterplot similar to the one in Figure 1. If the data points do not follow a linear pattern of relationship, but they appear to PS-related such as the patterns in the lower panel of Figure 1, the present study provides a good tool for the researchers to have a second thought and explore whether their data can be fitted to a PS-based SEM. The working example provides the details of how to execute the code, and this will open new era for behavioral researchers not only re-conceptualizing their causal models in CFA/SEM, but also potentially re-analyzing their CFA/SEM that may not show large effects (e.g., factor correlations, loadings, slopes, etc.) in previous research.

## Limitations and future directions

As the proposed PS-SEM is a relatively new concept, there are a number of limitations, leading to some future directions. First, this study simulates and evaluates only one CFA/SEM model based on DiStefano and Hess's (2005) review of 100 empirical SEM/CFA studies. As a starting point of PS-SEM, I believe that this is sufficient enough to show its potential in behavioral research. Future research can examine how the proposed PS-SEM estimator can be applied and used in complex CFA/SEM models (e.g., mediation/moderation, multi-level). Second, in light of the different assumption in PS-based CFA/SEM than the conventional linear-based CFA/SEM, the performance of the goodness-of-fit indices compromise under the proposed PS-based method. Additional research can explore and develop new goodness-of-fit indices that can be used and are more tailor-made in the framework of PS-SEM. Third, this study is an empirical, simulation-based study, which aims to provide empirical evidence of the potential of fitting a PS-based model in CFA/SEM. Future researchers can conduct a theoretical study that focuses on the mathematical proof of PS-based CFA/SEM, which can be formalized under the broader framework of generalized SEM (e.g., Skrondal and Rabe-Hesketh, 2004; Huber, 2013). Indeed, generalized SEM have included many other types of distribution of observed variables (e.g., binary, count, categorical, ordinal, censored continuous, survival, etc.), but no study, as I know, has attempted to incorporate the idea of how the observed variables are related based on the level of PS (i.e., PS-SEM) instead of distributions of scores (e.g., binary, categorical, etc.) under the generalized SEM. Given a more formal definition of PS-SEM under generalized SEM, researchers can better understand the future direction of the proposed PS-SEM, which in turn, providing a more complete picture about the sampling distributions and estimates of statistical coefficients (e.g., goodness-of-fit indices) in PS-SEM. Fourth, the present study only handles and focuses on simulated or real-world research scenarios with a full data-set. In the future, researchers can explore the use of other estimators (e.g., full information ML, multiple imputation) to model PS-based data with missing values.

## Author contributions

The author confirms being the sole contributor of this work and approved it for publication.

### Conflict of interest statement

The author declares that the research was conducted in the absence of any commercial or financial relationships that could be construed as a potential conflict of interest.

## References

[B1] Appelbaum M. Cooper H. Kline R. B. Mayo-Wilson E. Nezu A. M. Rao S. M. (2018). Journal article reporting standards for quantitative research in psychology: the APA Publications and Communications Board task force report. Am. Psychol. 73, 3–25. 10.1037/amp0000191 29345484

[B2] Bentler P. M. (1995). EQS Structural Equations Program Manual. Encino, CA: Multivariate Software.

[B3] Blomqvist N. (1950). On a measure of dependence between two random variables. Ann. Mathem. Stat. 21, 593–600. 10.1214/aoms/1177729754

[B4] Brooks M. E. Dalal D. K. Nolan K. P. (2014). Are common language effect sizes easier to understand than traditional effect sizes? J. Appl. Psychol. 99, 332–340. 10.1037/a0034745 24188393

[B5] Browne M. W. (1974). Generalized least-squares estimators in the analysis of covariance structures. South Afr. Stat. J. 8, 1–24.

[B6] Browne M. W. (1984). Asymptotically distribution-free methods for the analysis of covariance structures. Br. J. Mathem. Stat. Psychol. 37, 62–83. 10.1111/j.2044-8317.1984.tb00789.x 6733054

[B7] Byrne B. M. (2011). Structural Equation Modeling with Mplus: Basic Concepts, Applications, and Programming. New York, NY: Routledge.

[B8] Cheung M. W.-L. (2015). Meta-Analysis: A Structural Equation Modeling Approach. John Wiley & Sons, Ltd.

[B9] Cliff N. (1993). Dominance statistics: ordinal analyses to answer ordinal questions. Psychol. Bull. 114, 494–509. 10.1037/0033-2909.114.3.494

[B10] Danner D. Hagemann Fiedler (2015). Mediation analysis with structural equation models: combining theory, design, and statistics. Eur. J. Soc. Psychol. 45, 460–481. 10.1002/ejsp,.2106

[B11] DISC Personality Test (2018). DISC Overview. Available online at: https://www.discprofile.com/what-is-disc/overview/

[B12] DiStefano C. Hess B. (2005). Using confirmatory factor analysis for construct validation: an empirical review. J. Psychoeduc. Assess. 23, 225–241. 10.1177/073428290502300303

[B13] DiStefano C. Morgan G. B. (2014). A comparison of diagonal weighted least squares robust estimation techniques for ordinal data. Struct. Equ. Model. Multidisciplinary J. 21, 425–438. 10.1080/10705511.2014.915373

[B14] Dunlap W. P. (1994). Generalizing the common language effect size indicator to bivariate normal correlations. Psychol. Bull. 116, 509–511. 10.1037/0033-2909.116.3.509

[B15] Fan W. Hancock G. R. (2012). Robust means modeling: an alternative for hypothesis testing of independent means under variance heterogeneity and nonormality. J. Educ. Behav. Statist. 37, 137–156. 10.3102/1076998610396897

[B16] Grimm K. J. Ram N. Estabrook R. (2016). Growth Modeling – Structural Equation and Multilevel Modeling Approaches. New York, NY: The Guilford Press.

[B17] Hayduk L. A. (1987). Structural Equation Modeling with LISREL. Essentials and Advances. London, UK: Johns Hopkins Press Ltd.

[B18] Huber C. (2013). Generalized Structural Equation Modeling Using Stata, Italian Stata Users Group Meeting. Available online at: https://www.stata.com/meeting/italy13/abstracts/materials/it13_huber.pdf

[B19] Jackson D. L. Gillaspy J. A. Purc-Stephenson R. (2009). Reporting practices in confirmatory factor analysis: an overview and some recommendations. Psychol. Methods 14, 6–23. 10.1037/a0014694 19271845

[B20] Jöreskog K. G. Sörbom D. (1982). Recent developments in structural equation modeling. J. Market. Res. 19, 404–416. 10.2307/3151714

[B21] Kenny D. A. (2015). Measuring Model Fit. Available online at: http://davidakenny.net/cm/fit.htm

[B22] Kline R. B. (2015). Principles and Practice of Structural Equation Modeling (4th Edn). Guilford: NY.

[B23] Li J. C.-H. (2016). Effect size measures in a two independent-samples case with non-normal and non-homogeneous data. Behav. Res. Methods 48, 1560–1574. 10.3758/s13428-015-0667-z 26487051

[B24] Li J. C.-H. Chan W. Cui Y. (2011). Bootstrap standard error and confidence intervals for the correlations corrected for indirect range restriction. Br. J. Mathem. Stat. Psychol. 64, 367–387. 10.1348/2044-8317.002007 21973092

[B25] McGraw K. O. Wong S. P. (1992). A common language effect size statistic. Psychol. Bull. 111, 361–365. 10.1037/0033-2909.111.2.361

[B26] Newsom J. T. (2015). Longitudinal Structural Equation Modeling: A Comprehensive Introduction. New York, NY: Routledge.

[B27] Olsson U. H. Foss T. Troye S. V. Howell R. D. (2000). The performance of ML, GLS, and WLS estimation in structural equation modeling under conditions of misspecification and nonnormality. Struct. Equ. Model. 7, 557–595. 10.1207/S15328007SEM0704_3

[B28] Raw Data from Online Personality Tests (2018). Available online at: http://openpsychometrics.org/_rawdata/AS+SC+AD+DO.zip

[B29] Rosseel Y. (2012). lavaan: an R package for structural equation modeling. J. Stat. Softw. 48, 1–36. 10.18637/jss.v048.i02

[B30] R Studio Team (2017). RStudio: Integrated Development for R. Boston, MA: RStudio, Inc. Available online at: http://www.rstudio.com/

[B31] Ruscio J. (2008). A probability-based measure of effect size: robustness to base rates and other factors. Psychol. Methods 13, 19–30. 10.1037/1082-989X.13.1.19 18331151

[B32] Skrondal A. Rabe-Hesketh S. (2004). Generalized Latent Variable Modeling: Multilevel, Longitudinal and Structural Equation Models. Boca Raton, FL: Chapman & Hall/CRC.

[B33] Venables W. N. Ripley B. D. (2002). Modern Applied Statistics With S (4th Edn.). NewYork, NY: Springer.

[B34] Wolfe D. A. Hogg R. V. (1971). On constructing statistics and reporting data. Am. Stat. 25, 27–30.

