# Supplementary material for: Probability-of-Superiority SEM (PS-SEM)—Detecting Probability-Based Multivariate Relationships in Behavioral Research
Source: Front Psychol. 2018 Jun 13;9:883. doi: 10.3389/fpsyg.2018.00883 (PMC6008517; doi:10.3389/fpsyg.2018.00883)
Supplement: Supplementary file 1 [file Data_Sheet_1.DOCX]

**Supplementary Materials – Simulation Code**

require(MASS)

require(lavaan)

require(GenOrd)

library(distr)

library(truncdist)

library(GenOrd)

library(corpcor)

options(scipen=999)

pr<-function(data, cor = FALSE){

p <- dim(data)[2]

n <-length(data[,1])

re <- array(1,dim=c(p,p))

for(i in 1:p){

tem=i+1

if(tem<=p){

for(j in tem:p){

diff<-(data[,i]-mean(data[,i]))*(data[,j]-mean(data[,j]))

tcount<- length(as.integer(which(diff>0)))+length(as.integer(which(diff==0)))/2

re[i,j] <- tcount/n

}}}

ator <- function(a){return(sin(pi*(a-0.5)))}

f <- function(m) {

m[lower.tri(m)] <- t(m)[lower.tri(m)]

m

}

re <- ator(re)

re <- f(re)

if(cor==FALSE){

for(i in 1:p){

re[i,i] <- var(data[,i])

tem=i+1

if(tem<=p){

for(j in tem:p){

re[i,j] <- re[i,j]*sd(data[,i])*sd(data[,j])

}}}

re <- f(re)}

label <- colnames(data)

colnames(re) <- label

rownames(re) <- label

return(data.matrix(re))}

dir <- "C://" #directory for saving the output

#Simulation conditions

cond <- 1

set.seed(33883838)

replications <- 1000

rep <- 1

locpos <- c(3,4,5,9,17,18,19,20,22,23,26)

locpos2 <- c(6,7,8,25,32,33,34,35,37,44,47)

sdy <- sqrt(12)/2

k <- 20 #no. of items

mu <- rep(0,k)

rtoa <- function(r){return(asin(r)/pi+0.5)}

ator <- function(a){return(sin(pi*(a-0.5)))}

vb <- c(-2,-3,-5,-7,0,1,2,3,5,7) #-2 to -7: 2 point to 7 point linear-SEM, 0 = normal original, 1 = continuous uniform # 2 – 7 = PS-based 2 3 5 7 points

vn <- c(400,800,1200,1600) #sample size

vir <- c(.3) #factor correlation

vfload <- c(.7) #factor loading

tcond <- length(vb)*length(vn)*length(vir)*length(vfload)

cond.save <- array(0,dim=c(tcond,4))

cond.row <- character(length = tcond)

# Save results

fit.rep <- array(0, dim = c(tcond, (77*2)))

# pbm, ml,MLR, cont: decision chisqu p, cfi > .9, cfi > .95, rmsea <.08, rmesea < .05, rmsea p

dec.rep <- array(0, dim = c(tcond,(6*7)))

# paramater estimates

para.rep <- array(0, dim =c(tcond, (26*7*2)))

parase.rep <- array(0, dim =c(tcond, (26*7*2)))

# % bias, root mean squared error, mean absolute bias

bias.rep <- array(0, dim =c(tcond, (26*7*3)))

#save true parameter values

truepara.rep <- array(0, dim = c(tcond,26))

# of restart in each replication

restart.rep <- array(0, dim = c(tcond,5))

for(b.pos in 1:length(vb)){

b <- vb[b.pos]

for(n.pos in 1:length(vn)){

n <- vn[n.pos]

for(ir.pos in 1:length(vir)){

tir <- vir[ir.pos]

for(fload.pos in 1:length(vfload)){

tfload <- vfload[fload.pos]

fload <- tfload^2

ir <- tir*fload

cond.save[cond,] <- c(b,n,tir,tfload)

cond.row[cond] <- paste(as.character(cond.save[cond,]),collapse=", ")

true.para <- rep(c(rep(sqrt(fload),20),rep(ir/fload,6)),4)

truepara.rep[cond,] <- c(rep(sqrt(fload),20),rep(ir/fload,6))

#chisq. df. pvalue, cfi, logl, unrestricted.logl, aic, bic, bic2, rmsea, rmsea.pvalue

WLSpbm.fit <- array(NA, dim=c(replications,11))

MLpbm.fit <- array(NA, dim=c(replications,11))

ULSpbm.fit <- array(NA, dim=c(replications,11))

ML.fit <- array(NA, dim=c(replications,11))

MLR.fit <- array(NA, dim=c(replications,11))

ULS.fit<- array(NA, dim=c(replications,11))

WLS.fit <- array(NA, dim=c(replications,11))

all.fit <- array(NA, dim = c(replications, 77))

# save parameter estimates

WLSpbm.re <- array(NA, dim=c(replications,26))

MLpbm.re <- array(NA, dim=c(replications,26))

ULSpbm.re <- array(NA, dim=c(replications,26))

ML.re<- array(NA, dim=c(replications,26))

MLR.re<- array(NA, dim=c(replications,26))

ULS.re<- array(NA, dim=c(replications,26))

WLS.re<- array(NA, dim=c(replications,26))

all.re <- array(NA, dim = c(replications, (26*7)))

WLSpbm.rese <- array(NA, dim=c(replications,26))

MLpbm.rese <- array(NA, dim=c(replications,26))

ULSpbm.rese <- array(NA, dim=c(replications,26))

ML.rese<- array(NA, dim=c(replications,26))

MLR.rese<- array(NA, dim=c(replications,26))

ULS.rese<- array(NA, dim=c(replications,26))

WLS.rese<- array(NA, dim=c(replications,26))

all.rese <- array(NA, dim = c(replications, (26*7)))

tcov <- matrix(c(1,fload,fload,fload,fload,ir,ir,ir,ir,ir,ir,ir,ir,ir,ir,ir,ir,ir,ir,ir,

fload,1,fload,fload,fload,ir,ir,ir,ir,ir,ir,ir,ir,ir,ir,ir,ir,ir,ir,ir,

fload,fload,1,fload,fload,ir,ir,ir,ir,ir,ir,ir,ir,ir,ir,ir,ir,ir,ir,ir,

fload,fload,fload,1,fload,ir,ir,ir,ir,ir,ir,ir,ir,ir,ir,ir,ir,ir,ir,ir,

fload,fload,fload,fload,1,ir,ir,ir,ir,ir,ir,ir,ir,ir,ir,ir,ir,ir,ir,ir,

ir,ir,ir,ir,ir,1,fload,fload,fload,fload,ir,ir,ir,ir,ir,ir,ir,ir,ir,ir,

ir,ir,ir,ir,ir,fload,1,fload,fload,fload,ir,ir,ir,ir,ir,ir,ir,ir,ir,ir,

ir,ir,ir,ir,ir,fload,fload,1,fload,fload,ir,ir,ir,ir,ir,ir,ir,ir,ir,ir,

ir,ir,ir,ir,ir,fload,fload,fload,1,fload,ir,ir,ir,ir,ir,ir,ir,ir,ir,ir,

ir,ir,ir,ir,ir,fload,fload,fload,fload,1,ir,ir,ir,ir,ir,ir,ir,ir,ir,ir,

ir,ir,ir,ir,ir,ir,ir,ir,ir,ir,1,fload,fload,fload,fload,ir,ir,ir,ir,ir,

ir,ir,ir,ir,ir,ir,ir,ir,ir,ir,fload,1,fload,fload,fload,ir,ir,ir,ir,ir,

ir,ir,ir,ir,ir,ir,ir,ir,ir,ir,fload,fload,1,fload,fload,ir,ir,ir,ir,ir,

ir,ir,ir,ir,ir,ir,ir,ir,ir,ir,fload,fload,fload,1,fload,ir,ir,ir,ir,ir,

ir,ir,ir,ir,ir,ir,ir,ir,ir,ir,fload,fload,fload,fload,1,ir,ir,ir,ir,ir,

ir,ir,ir,ir,ir,ir,ir,ir,ir,ir,ir,ir,ir,ir,ir,1,fload,fload,fload,fload,

ir,ir,ir,ir,ir,ir,ir,ir,ir,ir,ir,ir,ir,ir,ir,fload,1,fload,fload,fload,

ir,ir,ir,ir,ir,ir,ir,ir,ir,ir,ir,ir,ir,ir,ir,fload,fload,1,fload,fload,

ir,ir,ir,ir,ir,ir,ir,ir,ir,ir,ir,ir,ir,ir,ir,fload,fload,fload,1,fload,

ir,ir,ir,ir,ir,ir,ir,ir,ir,ir,ir,ir,ir,ir,ir,fload,fload,fload,fload,1),k,k)

for(rep in 1:replications){

restart1 <- 1

while(restart1>0){

restart1 <- 0

if(b>=0){

rdat <- mvrnorm(n,mu,tcov)}else{

if(b==-2){

marginal <- rep(list(c(0.5)),20)

rdat <- ordsample(n,marginal,tcov)}

if(b==-3){

marginal <- rep(list(c(0.2,0.8)),20)

rdat <- ordsample(n,marginal,tcov)}

if(b==-5){

marginal <- rep(list(c(0.1,0.3,0.7,0.9)),20)

rdat <- ordsample(n,marginal,tcov)}

if(b==-7){

marginal <- rep(list(c(0.05,0.15,0.35,0.65,0.85,0.95)),20)

rdat <- ordsample(n,marginal,tcov)}

}#end of b < 0

pdat <- rdat

colnames(pdat) <- c("x1","x2","x3","x4","x5","x6","x7","x8","x9","x10","x11","x12","x13","x14","x15","x16","x17","x18","x19","x20")

colnames(rdat) <- c("x1","x2","x3","x4","x5","x6","x7","x8","x9","x10","x11","x12","x13","x14","x15","x16","x17","x18","x19","x20")

pdat <- data.frame(pdat)

rdat <- data.frame(rdat)

psign <- array(0, dim=c(n,k))

for(i in 1:k){

psign[which(rdat[,i]>mean(rdat[,i])),i] <- 1}

if(b==1){

for(i in 1:n){

for(j in 1:k){

if(psign[i,j]==1){pdat[i,j] <- runif(1,0,sdy)}else{pdat[i,j] <- runif(1,-sdy,0)}}}}

if(b>1){

m <- b/2+0.1

if(b>2){

for(i in 1:n){

for(j in 1:k){

if(psign[i,j]==1){pdat[i,j] <- sample(ceiling(m):b,1)}

else{pdat[i,j] <- sample(1:floor(m),1) }}}}

if(b==2){for(i in 1:n){

for(j in 1:k){

if(psign[i,j]==1){pdat[i,j] <- 1}

else{pdat[i,j] <- 0 }}}}}

samcov <- pr(pdat)

tsamcov <- pr(pdat,cor=TRUE)

colnames(samcov) <- c("x1","x2","x3","x4","x5","x6","x7","x8","x9","x10","x11","x12","x13","x14","x15","x16","x17","x18","x19","x20")

colnames(samcov) <- c("x1","x2","x3","x4","x5","x6","x7","x8","x9","x10","x11","x12","x13","x14","x15","x16","x17","x18","x19","x20")

HS.model <- ' F1 =~ x1 + x2 + x3 + x4 + x5

F2 =~ x6 + x7 + x8 + x9 + x10

F3 =~ x11 + x12 + x13 + x14 + x15

F4 =~ x16 + x17 + x18 + x19 + x20'

# check positive non definite covariance matrix

check1 <- tryCatch(cfa(HS.model, sample.cov = samcov, sample.nobs = n),

error=function(e) e,

warning=function(w) w)

if(class(check1)[[1]]=="simpleWarning"){restart1 <- 1

restart.rep[cond,1]<-restart.rep[cond,1]+1}

# Function to obtain the weighted matrix for PS-WLS

#trep <- 10

#tWLS.V <- array(0, dim=c(trep,210,210))

#for(i in 1:trep){

#wdat <- mvrnorm(n,mu,pr(pdat,cor=TRUE))

# tfit <- cfa(HS.model, data=wdat, std.lv=TRUE, estimator = "WLS")

# tWLS.V[i,,] <- inspect(tfit, "WLS.V")

#}

#weightmatrix <- apply(tWLS.V, c(2,3), function(x) mean(x))

omu <- array(0, dim=c(20))

for(j in 1:20){

omu[j] <- mean(pdat[,j])}

check4 <- is.positive.definite(samcov)

if(check4 == TRUE){

wdat <- mvrnorm(20000,mu,samcov)

tfit <- cfa(HS.model, data=wdat, estimator = "WLS")

weightmatrix <- inspect(tfit, "WLS.V")

}else{restart1 <- 1

restart.rep[cond,4]<-restart.rep[cond,4]+1}

# check positive non definite covariance matrix

check2 <- tryCatch(cfa(HS.model, estimator="WLS", WLS.V=weightmatrix, sample.cov = samcov, sample.nobs = n),

error=function(e) e,

warning=function(w) w)

if(class(check2)[[1]]=="simpleWarning"){restart1 <- 1

restart.rep[cond,2]<-restart.rep[cond,2]+1}

check3 <- tryCatch(cfa(HS.model, estimator = "WLS", data = pdat),

error=function(e) e,

warning=function(w) w)

if(class(check3)[[1]]=="simpleError"){restart1 <- 1

restart.rep[cond,3]<-restart.rep[cond,3]+1}

check5 <- tryCatch(cfa(HS.model, estimator="ULS", sample.cov = samcov, sample.nobs = n),

error=function(e) e,

warning=function(w) w)

if(class(check5)[[1]]=="simpleWarning"){restart1 <- 1

restart.rep[cond,5]<-restart.rep[cond,5]+1}

}

###################################################

tWLSpbm.fit <- cfa(HS.model, estimator="WLS", WLS.V=weightmatrix, sample.cov = samcov, sample.nobs = n)

WLSpbm.fit[rep,] <- fitMeasures(tWLSpbm.fit)[locpos]

tMLpbm.fit <- cfa(HS.model, estimator="ML", sample.cov = samcov, sample.nobs = n)

MLpbm.fit[rep,] <- fitMeasures(tMLpbm.fit)[locpos]

tULSpbm.fit <- cfa(HS.model, estimator="ULS", sample.cov = samcov, sample.nobs = n)

ULSpbm.fit[rep,] <- fitMeasures(tULSpbm.fit)[locpos]

tML.fit <- cfa(HS.model, data = pdat, estimator = "ML")

ML.fit[rep,] <- fitMeasures(tML.fit)[locpos]

tMLR.fit <- cfa(HS.model, estimator = "MLR", data = pdat)

MLR.fit[rep,] <- fitMeasures(tMLR.fit)[locpos2]

tULS.fit <- cfa(HS.model, estimator = "ULS", data = pdat)

ULS.fit[rep,] <- fitMeasures(tULS.fit)[locpos]

tWLS.fit <- cfa(HS.model, estimator = "WLS", data = pdat)

WLS.fit[rep,] <- fitMeasures(tWLS.fit)[locpos]

ULSpbm.fit[rep,3] <- 1

ULS.fit[rep,3] <- 1

#tcont.fit <- cfa(HS.model, data = rdat)

#cont.fit[rep,] <- fitMeasures(tcont.fit)[locpos]

all.fit[rep,] <- c(WLSpbm.fit[rep,],MLpbm.fit[rep,],ULSpbm.fit[rep,],

ML.fit[rep,],MLR.fit[rep,],WLS.fit[rep,],ULS.fit[rep,])

WLSpbm.re[rep,] <- c(standardizedSolution(tWLSpbm.fit)[[4]][1:20],standardizedSolution(tWLSpbm.fit)[[4]][45:50])

MLpbm.re[rep,] <- c(standardizedSolution(tMLpbm.fit)[[4]][1:20],standardizedSolution(tMLpbm.fit)[[4]][45:50])

ULSpbm.re[rep,] <- c(standardizedSolution(tULSpbm.fit)[[4]][1:20],standardizedSolution(tULSpbm.fit)[[4]][45:50])

ML.re[rep,] <- c(standardizedSolution(tML.fit)[[4]][1:20],standardizedSolution(tML.fit)[[4]][45:50])

MLR.re[rep,] <- c(standardizedSolution(tMLR.fit)[[4]][1:20],standardizedSolution(tMLR.fit)[[4]][45:50])

ULS.re[rep,] <- c(standardizedSolution(tULS.fit)[[4]][1:20],standardizedSolution(tULS.fit)[[4]][45:50])

WLS.re[rep,] <- c(standardizedSolution(tWLS.fit)[[4]][1:20],standardizedSolution(tWLS.fit)[[4]][45:50])

all.re[rep,] <- c(WLSpbm.re[rep,],MLpbm.re[rep,],ULSpbm.re[rep,],ML.re[rep,],MLR.re[rep,],ULS.re[rep,],WLS.re[rep,] )

WLSpbm.rese[rep,] <- c(standardizedSolution(tWLSpbm.fit)[[5]][1:20],standardizedSolution(tWLSpbm.fit)[[5]][45:50])

MLpbm.rese[rep,] <- c(standardizedSolution(tMLpbm.fit)[[5]][1:20],standardizedSolution(tMLpbm.fit)[[5]][45:50])

ULSpbm.rese[rep,] <- c(standardizedSolution(tULSpbm.fit)[[5]][1:20],standardizedSolution(tULSpbm.fit)[[5]][45:50])

ML.rese[rep,] <- c(standardizedSolution(tML.fit)[[5]][1:20],standardizedSolution(tML.fit)[[5]][45:50])

MLR.rese[rep,] <- c(standardizedSolution(tMLR.fit)[[5]][1:20],standardizedSolution(tMLR.fit)[[5]][45:50])

ULS.rese[rep,] <- c(standardizedSolution(tULS.fit)[[5]][1:20],standardizedSolution(tULS.fit)[[5]][45:50])

WLS.rese[rep,] <- c(standardizedSolution(tWLS.fit)[[5]][1:20],standardizedSolution(tWLS.fit)[[5]][45:50])

all.rese[rep,] <- c(WLSpbm.rese[rep,],MLpbm.rese[rep,],ULSpbm.rese[rep,],ML.rese[rep,],MLR.rese[rep,],ULS.rese[rep,],WLS.rese[rep,] )

# fit testing

#chisq. df. pvalue, cfi, logl, unrestricted.logl, aic, bic, bic2, rmsea, rmsea.pvalue

# pbm, ml, mlr, cont: decision chisqu p, cfi > .9, cfi > .95, rmsea <.08, rmesea < .05, rmsea p

if(WLSpbm.fit[rep,3]>.05){dec.rep[cond,1] <- dec.rep[cond,1]+1}

if(WLSpbm.fit[rep,4]>.9){dec.rep[cond,2] <- dec.rep[cond,2]+1}

if(WLSpbm.fit[rep,4]>.95){dec.rep[cond,3] <- dec.rep[cond,3]+1}

if(WLSpbm.fit[rep,10]<.08){dec.rep[cond,4] <- dec.rep[cond,4]+1}

if(WLSpbm.fit[rep,10]<.05){dec.rep[cond,5] <- dec.rep[cond,5]+1}

if(WLSpbm.fit[rep,11]>.05){dec.rep[cond,6] <- dec.rep[cond,6]+1}

if(MLpbm.re[rep,3]>.05){dec.rep[cond,7] <- dec.rep[cond,7]+1}

if(MLpbm.re[rep,4]>.9){dec.rep[cond,8] <- dec.rep[cond,8]+1}

if(MLpbm.re[rep,4]>.95){dec.rep[cond,9] <- dec.rep[cond,9]+1}

if(MLpbm.re[rep,10]<.08){dec.rep[cond,10] <- dec.rep[cond,10]+1}

if(MLpbm.re[rep,10]<.05){dec.rep[cond,11] <- dec.rep[cond,11]+1}

if(MLpbm.re[rep,11]>.05){dec.rep[cond,12] <- dec.rep[cond,12]+1}

if(ULSpbm.re[rep,3]>.05){dec.rep[cond,13] <- dec.rep[cond,13]+1}

if(ULSpbm.re[rep,4]>.9){dec.rep[cond,14] <- dec.rep[cond,14]+1}

if(ULSpbm.re[rep,4]>.95){dec.rep[cond,15] <- dec.rep[cond,15]+1}

if(ULSpbm.re[rep,10]<.08){dec.rep[cond,16] <- dec.rep[cond,16]+1}

if(ULSpbm.re[rep,10]<.05){dec.rep[cond,17] <- dec.rep[cond,17]+1}

if(ULSpbm.re[rep,11]>.05){dec.rep[cond,18] <- dec.rep[cond,18]+1}

if(ML.re[rep,3]>.05){dec.rep[cond,19] <- dec.rep[cond,19]+1}

if(ML.re[rep,4]>.9){dec.rep[cond,20] <- dec.rep[cond,20]+1}

if(ML.re[rep,4]>.95){dec.rep[cond,21] <- dec.rep[cond,21]+1}

if(ML.re[rep,10]<.08){dec.rep[cond,22] <- dec.rep[cond,22]+1}

if(ML.re[rep,10]<.05){dec.rep[cond,23] <- dec.rep[cond,23]+1}

if(ML.re[rep,11]>.05){dec.rep[cond,24] <- dec.rep[cond,24]+1}

if(MLR.re[rep,3]>.05){dec.rep[cond,25] <- dec.rep[cond,25]+1}

if(MLR.re[rep,4]>.9){dec.rep[cond,26] <- dec.rep[cond,26]+1}

if(MLR.re[rep,4]>.95){dec.rep[cond,27] <- dec.rep[cond,27]+1}

if(MLR.re[rep,10]<.08){dec.rep[cond,28] <- dec.rep[cond,28]+1}

if(MLR.re[rep,10]<.05){dec.rep[cond,29] <- dec.rep[cond,29]+1}

if(MLR.re[rep,11]>.05){dec.rep[cond,30] <- dec.rep[cond,30]+1}

if(ULS.re[rep,3]>.05){dec.rep[cond,31] <- dec.rep[cond,31]+1}

if(ULS.re[rep,4]>.9){dec.rep[cond,32] <- dec.rep[cond,32]+1}

if(ULS.re[rep,4]>.95){dec.rep[cond,33] <- dec.rep[cond,33]+1}

if(ULS.re[rep,10]<.08){dec.rep[cond,34] <- dec.rep[cond,34]+1}

if(ULS.re[rep,10]<.05){dec.rep[cond,35] <- dec.rep[cond,35]+1}

if(ULS.re[rep,11]>.05){dec.rep[cond,36] <- dec.rep[cond,36]+1}

if(WLS.re[rep,3]>.05){dec.rep[cond,37] <- dec.rep[cond,37]+1}

if(WLS.re[rep,4]>.9){dec.rep[cond,38] <- dec.rep[cond,38]+1}

if(WLS.re[rep,4]>.95){dec.rep[cond,39] <- dec.rep[cond,39]+1}

if(WLS.re[rep,10]<.08){dec.rep[cond,40] <- dec.rep[cond,40]+1}

if(WLS.re[rep,10]<.05){dec.rep[cond,41] <- dec.rep[cond,41]+1}

if(WLS.re[rep,11]>.05){dec.rep[cond,42] <- dec.rep[cond,42]+1}

} # end of replications

# mean and sd of fit indices

for(i in 1:77){

fit.rep[cond,i] <- mean(all.fit[,i])

fit.rep[cond,(i+77)] <- sd(all.fit[,i])}

# mean and sd of parameter estimates

for(i in 1:(26*7)){

para.rep[cond,i] <-mean(all.re[,i])

para.rep[cond,(i+182)] <-sd(all.re[,i])

parase.rep[cond,i] <-mean(all.rese[,i])

parase.rep[cond,(i+182)] <-sd(all.rese[,i])

}

# % bias, root mean squared error, mean absolute bias

for(i in 1:182){

bias.rep[cond,i] <- (para.rep[cond,i]-true.para[i])/true.para[i]

bias.rep[cond,(i+182)] <- sqrt(sum((all.re[,i]-true.para[i])^2)/replications)

bias.rep[cond,(i+364)] <- sum(abs(all.re[,i]-true.para[i]))/replications}

#save intermediate output

if((cond/10)%%1==0){

write.table(fit.rep, paste(dir,"BUfit.csv",sep=""),col.names=NA,sep=",")

write.table(dec.rep, paste(dir,"BUdecision.csv",sep=""),col.names=NA,sep=",")

write.table(para.rep, paste(dir,"BUparameters.csv",sep=""),col.names=NA,sep=",")

write.table(bias.rep, paste(dir,"BUbias.csv",sep=""),col.names=NA,sep=",")

write.table(cond.save, paste(dir,"BUconditions.csv",sep=""),col.names=NA,sep=",")

write.table(truepara.rep, paste(dir,"BUtrueparameters.csv",sep=""),row.names=FALSE,col.names=FALSE,sep=",")

write.table(restart.rep, paste(dir,"BUrestart.csv",sep=""),col.names=NA,sep=",")

}

write.table(cond, paste(dir,"cond.csv",sep=""),col.names=NA,sep=",")

cond <- cond+1}}}} #end of conditions

#Export data

colnames(cond.save) <- c("b (no. of points)","n","ir","fload")

col1 <- c("mean WLSpb chisq", "mean WLSpb df", "mean WLSpb chi pvalue", "mean WLSpb cfi", "mean WLSpb logl", "mean WLSpb unrestricted.logl",

"mean WLSpb aic", "mean WLSpb bic", "mean WLSpb bic2", "mean WLSpb rmsea", "mean WLSpb rmsea.pvalue",

"mean MLpb chisq", "mean MLpb df", "mean MLpb chi pvalue", "mean MLpb cfi", "mean MLpb logl", "mean MLpb unrestricted.logl",

"mean MLpb aic", "mean MLpb bic", "mean MLpb bic2", "mean MLpb rmsea", "mean MLpb rmsea.pvalue",

"mean ULSpb chisq", "mean ULSpb df", "mean ULSpb chi pvalue", "mean ULSpb cfi", "mean ULSpb logl", "mean ULSpb unrestricted.logl",

"mean ULSpb aic", "mean ULSpb bic", "mean ULSpb bic2", "mean ULSpb rmsea", "mean ULSpb rmsea.pvalue",

"mean ML chisq", "mean ML df", "mean ML chi pvalue", "mean ML cfi", "mean ML logl", "mean ML unrestricted.logl",

"mean ML aic", "mean ML bic", "mean ML bic2", "mean ML rmsea", "mean ML rmsea.pvalue",

"mean MLR chisq", "mean MLR df", "mean MLR chi pvalue", "mean MLR cfi", "mean MLR logl", "mean MLR unrestricted.logl",

"mean MLR aic", "mean MLR bic", "mean MLR bic2", "mean MLR rmsea", "mean MLR rmsea.pvalue",

"mean ULS chisq", "mean ULS df", "mean ULS chi pvalue", "mean ULS cfi", "mean ULS logl", "mean ULS unrestricted.logl",

"mean ULS aic", "mean ULS bic", "mean ULS bic2", "mean ULS rmsea", "mean ULS rmsea.pvalue",

"mean WLS chisq", "mean WLS df", "mean WLS chi pvalue", "mean WLS cfi", "mean WLS logl", "mean WLS unrestricted.logl",

"mean WLS aic", "mean WLS bic", "mean WLS bic2", "mean WLS rmsea", "mean WLS rmsea.pvalue",

"sd WLSpb chisq", "sd WLSpb df", "sd WLSpb chi pvalue", "sd WLSpb cfi", "sd WLSpb logl", "sd WLSpb unrestricted.logl",

"sd WLSpb aic", "sd WLSpb bic", "sd WLSpb bic2", "sd WLSpb rmsea", "sd WLSpb rmsea.pvalue",

"sd MLpb chisq", "sd MLpb df", "sd MLpb chi pvalue", "sd MLpb cfi", "sd MLpb logl", "sd MLpb unrestricted.logl",

"sd MLpb aic", "sd MLpb bic", "sd MLpb bic2", "sd MLpb rmsea", "sd MLpb rmsea.pvalue",

"sd ULSpb chisq", "sd ULSpb df", "sd ULSpb chi pvalue", "sd ULSpb cfi", "sd ULSpb logl", "sd ULSpb unrestricted.logl",

"sd ULSpb aic", "sd ULSpb bic", "sd ULSpb bic2", "sd ULSpb rmsea", "sd ULSpb rmsea.pvalue",

"sd ML chisq", "sd ML df", "sd ML chi pvalue", "sd ML cfi", "sd ML logl", "sd ML unrestricted.logl",

"sd ML aic", "sd ML bic", "sd ML bic2", "sd ML rmsea", "sd ML rmsea.pvalue",

"sd MLR chisq", "sd MLR df", "sd MLR chi pvalue", "sd MLR cfi", "sd MLR logl", "sd MLR unrestricted.logl",

"sd MLR aic", "sd MLR bic", "sd MLR bic2", "sd MLR rmsea", "sd MLR rmsea.pvalue",

"sd ULS chisq", "sd ULS df", "sd ULS chi pvalue", "sd ULS cfi", "sd ULS logl", "sd ULS unrestricted.logl",

"sd ULS aic", "sd ULS bic", "sd ULS bic2", "sd ULS rmsea", "sd ULS rmsea.pvalue",

"sd WLS chisq", "sd WLS df", "sd WLS chi pvalue", "sd WLS cfi", "sd WLS logl", "sd WLS unrestricted.logl",

"sd WLS aic", "sd WLS bic", "sd WLS bic2", "sd WLS rmsea", "sd WLS rmsea.pvalue")

colnames(fit.rep) <- col1

col2 <- c("WLSpb chisqu p", "WLSpb cfi .9", "WLSpb cfi .95", "WLSpb rmsea .08", "WLSpb rmsea .05", "WLSpb rmsea p",

"MLpb chisqu p", "MLpb cfi .9", "MLpb cfi .95", "MLpb rmsea .08", "MLpb rmsea .05", "MLpb rmsea p",

"ULSpb chisqu p", "ULSpb cfi .9", "ULSpb cfi .95", "ULSpb rmsea .08", "ULSpb rmsea .05", "ULSpb rmsea p",

"ML chisqu p", "ML cfi .9", "ML cfi .95", "ML rmsea .08", "ML rmsea .05", "ML rmsea p",

"MLR chisqu p", "MLR cfi .9", "MLR cfi .95", "MLR rmsea .08", "MLR rmsea .05", "MLR rmsea p",

"ULS chisqu p", "ULS cfi .9", "ULS cfi .95", "ULS rmsea .08", "ULS rmsea .05", "ULS rmsea p",

"WLS chisqu p", "WLS cfi .9", "WLS cfi .95", "WLS rmsea .08", "WLS rmsea .05", "WLS rmsea p")

colnames(dec.rep) <- col2

col4 <- c("mean WLSpb y1","mean WLSpb y2","mean WLSpb y3","mean WLSpb y4","mean WLSpb y5",

"mean WLSpb y6","mean WLSpb y7","mean WLSpb y8","mean WLSpb y9","mean WLSpb y10",

"mean WLSpb y11","mean WLSpb y12","mean WLSpb y13","mean WLSpb y14","mean WLSpb y15",

"mean WLSpb y16","mean WLSpb y17","mean WLSpb 18","mean WLSpb y19","mean WLSpb y20",

"mean WLSpb f12","mean WLSpb f13","mean WLSpb f14","mean WLSpb f23","mean WLSpb f24","mean WLSpb f34",

"mean MLpb y1","mean MLpb y2","mean MLpb y3","mean MLpb y4","mean MLpb y5",

"mean MLpb y6","mean MLpb y7","mean MLpb y8","mean MLpb y9","mean MLpb y10",

"mean MLpb y11","mean MLpb y12","mean MLpb y13","mean MLpb y14","mean MLpb y15",

"mean MLpb y16","mean MLpb y17","mean MLpb 18","mean MLpb y19","mean MLpb y20",

"mean MLpb f12","mean MLpb f13","mean MLpb f14","mean MLpb f23","mean MLpb f24","mean MLpb f34",

"mean ULSpb y1","mean ULSpb y2","mean ULSpb y3","mean ULSpb y4","mean ULSpb y5",

"mean ULSpb y6","mean ULSpb y7","mean ULSpb y8","mean ULSpb y9","mean ULSpb y10",

"mean ULSpb y11","mean ULSpb y12","mean ULSpb y13","mean ULSpb y14","mean ULSpb y15",

"mean ULSpb y16","mean ULSpb y17","mean ULSpb 18","mean ULSpb y19","mean ULSpb y20",

"mean ULSpb f12","mean ULSpb f13","mean ULSpb f14","mean ULSpb f23","mean ULSpb f24","mean ULSpb f34",

"mean ML y1","mean ML y2","mean ML y3","mean ML y4","mean ML y5",

"mean ML y6","mean ML y7","mean ML y8","mean ML y9","mean ML y10",

"mean ML y11","mean ML y12","mean ML y13","mean ML y14","mean ML y15",

"mean ML y16","mean ML y17","mean ML 18","mean ML y19","mean ML y20",

"mean ML f12","mean ML f13","mean ML f14","mean ML f23","mean ML f24","mean ML f34",

"mean MLR y1","mean MLR y2","mean MLR y3","mean MLR y4","mean MLR y5",

"mean MLR y6","mean MLR y7","mean MLR y8","mean MLR y9","mean MLR y10",

"mean MLR y11","mean MLR y12","mean MLR y13","mean MLR y14","mean MLR y15",

"mean MLR y16","mean MLR y17","mean MLR 18","mean MLR y19","mean MLR y20",

"mean MLR f12","mean MLR f13","mean MLR f14","mean MLR f23","mean MLR f24","mean MLR f34",

"mean ULS y1","mean ULS y2","mean ULS y3","mean ULS y4","mean ULS y5",

"mean ULS y6","mean ULS y7","mean ULS y8","mean ULS y9","mean ULS y10",

"mean ULS y11","mean ULS y12","mean ULS y13","mean ULS y14","mean ULS y15",

"mean ULS y16","mean ULS y17","mean ULS 18","mean ULS y19","mean ULS y20",

"mean ULS f12","mean ULS f13","mean ULS f14","mean ULS f23","mean ULS f24","mean ULS f34",

"mean WLS y1","mean WLS y2","mean WLS y3","mean WLS y4","mean WLS y5",

"mean WLS y6","mean WLS y7","mean WLS y8","mean WLS y9","mean WLS y10",

"mean WLS y11","mean WLS y12","mean WLS y13","mean WLS y14","mean WLS y15",

"mean WLS y16","mean WLS y17","mean WLS 18","mean WLS y19","mean WLS y20",

"mean WLS f12","mean WLS f13","mean WLS f14","mean WLS f23","mean WLS f24","mean WLS f34",

"sd WLSpb y1","sd WLSpb y2","sd WLSpb y3","sd WLSpb y4","sd WLSpb y5",

"sd WLSpb y6","sd WLSpb y7","sd WLSpb y8","sd WLSpb y9","sd WLSpb y10",

"sd WLSpb y11","sd WLSpb y12","sd WLSpb y13","sd WLSpb y14","sd WLSpb y15",

"sd WLSpb y16","sd WLSpb y17","sd WLSpb 18","sd WLSpb y19","sd WLSpb y20",

"sd WLSpb f12","sd WLSpb f13","sd WLSpb f14","sd WLSpb f23","sd WLSpb f24","sd WLSpb f34",

"sd MLpb y1","sd MLpb y2","sd MLpb y3","sd MLpb y4","sd MLpb y5",

"sd MLpb y6","sd MLpb y7","sd MLpb y8","sd MLpb y9","sd MLpb y10",

"sd MLpb y11","sd MLpb y12","sd MLpb y13","sd MLpb y14","sd MLpb y15",

"sd MLpb y16","sd MLpb y17","sd MLpb 18","sd MLpb y19","sd MLpb y20",

"sd MLpb f12","sd MLpb f13","sd MLpb f14","sd MLpb f23","sd MLpb f24","sd MLpb f34",

"sd ULSpb y1","sd ULSpb y2","sd ULSpb y3","sd ULSpb y4","sd ULSpb y5",

"sd ULSpb y6","sd ULSpb y7","sd ULSpb y8","sd ULSpb y9","sd ULSpb y10",

"sd ULSpb y11","sd ULSpb y12","sd ULSpb y13","sd ULSpb y14","sd ULSpb y15",

"sd ULSpb y16","sd ULSpb y17","sd ULSpb 18","sd ULSpb y19","sd ULSpb y20",

"sd ULSpb f12","sd ULSpb f13","sd ULSpb f14","sd ULSpb f23","sd ULSpb f24","sd ULSpb f34",

"sd ML y1","sd ML y2","sd ML y3","sd ML y4","sd ML y5",

"sd ML y6","sd ML y7","sd ML y8","sd ML y9","sd ML y10",

"sd ML y11","sd ML y12","sd ML y13","sd ML y14","sd ML y15",

"sd ML y16","sd ML y17","sd ML 18","sd ML y19","sd ML y20",

"sd ML f12","sd ML f13","sd ML f14","sd ML f23","sd ML f24","sd ML f34",

"sd MLR y1","sd MLR y2","sd MLR y3","sd MLR y4","sd MLR y5",

"sd MLR y6","sd MLR y7","sd MLR y8","sd MLR y9","sd MLR y10",

"sd MLR y11","sd MLR y12","sd MLR y13","sd MLR y14","sd MLR y15",

"sd MLR y16","sd MLR y17","sd MLR 18","sd MLR y19","sd MLR y20",

"sd MLR f12","sd MLR f13","sd MLR f14","sd MLR f23","sd MLR f24","sd MLR f34",

"sd ULS y1","sd ULS y2","sd ULS y3","sd ULS y4","sd ULS y5",

"sd ULS y6","sd ULS y7","sd ULS y8","sd ULS y9","sd ULS y10",

"sd ULS y11","sd ULS y12","sd ULS y13","sd ULS y14","sd ULS y15",

"sd ULS y16","sd ULS y17","sd ULS 18","sd ULS y19","sd ULS y20",

"sd ULS f12","sd ULS f13","sd ULS f14","sd ULS f23","sd ULS f24","sd ULS f34",

"sd WLS y1","sd WLS y2","sd WLS y3","sd WLS y4","sd WLS y5",

"sd WLS y6","sd WLS y7","sd WLS y8","sd WLS y9","sd WLS y10",

"sd WLS y11","sd WLS y12","sd WLS y13","sd WLS y14","sd WLS y15",

"sd WLS y16","sd WLS y17","sd WLS 18","sd WLS y19","sd WLS y20",

"sd WLS f12","sd WLS f13","sd WLS f14","sd WLS f23","sd WLS f24","sd WLS f34")

colnames(para.rep) <- col4

colnames(parase.rep) <- col4

col5 <- c("% bias WLSpb y1","% bias WLSpb y2","% bias WLSpb y3","% bias WLSpb y4","% bias WLSpb y5",

"% bias WLSpb y6","% bias WLSpb y7","% bias WLSpb y8","% bias WLSpb y9","% bias WLSpb y10",

"% bias WLSpb y11","% bias WLSpb y12","% bias WLSpb y13","% bias WLSpb y14","% bias WLSpb y15",

"% bias WLSpb y16","% bias WLSpb y17","% bias WLSpb 18","% bias WLSpb y19","% bias WLSpb y20",

"% bias WLSpb f12","% bias WLSpb f13","% bias WLSpb f14","% bias WLSpb f23","% bias WLSpb f24","% bias WLSpb f34",

"% bias MLpb y1","% bias MLpb y2","% bias MLpb y3","% bias MLpb y4","% bias MLpb y5",

"% bias MLpb y6","% bias MLpb y7","% bias MLpb y8","% bias MLpb y9","% bias MLpb y10",

"% bias MLpb y11","% bias MLpb y12","% bias MLpb y13","% bias MLpb y14","% bias MLpb y15",

"% bias MLpb y16","% bias MLpb y17","% bias MLpb 18","% bias MLpb y19","% bias MLpb y20",

"% bias MLpb f12","% bias MLpb f13","% bias MLpb f14","% bias MLpb f23","% bias MLpb f24","% bias MLpb f34",

"% bias ULSpb y1","% bias ULSpb y2","% bias ULSpb y3","% bias ULSpb y4","% bias ULSpb y5",

"% bias ULSpb y6","% bias ULSpb y7","% bias ULSpb y8","% bias ULSpb y9","% bias ULSpb y10",

"% bias ULSpb y11","% bias ULSpb y12","% bias ULSpb y13","% bias ULSpb y14","% bias ULSpb y15",

"% bias ULSpb y16","% bias ULSpb y17","% bias ULSpb 18","% bias ULSpb y19","% bias ULSpb y20",

"% bias ULSpb f12","% bias ULSpb f13","% bias ULSpb f14","% bias ULSpb f23","% bias ULSpb f24","% bias ULSpb f34",

"% bias ML y1","% bias ML y2","% bias ML y3","% bias ML y4","% bias ML y5",

"% bias ML y6","% bias ML y7","% bias ML y8","% bias ML y9","% bias ML y10",

"% bias ML y11","% bias ML y12","% bias ML y13","% bias ML y14","% bias ML y15",

"% bias ML y16","% bias ML y17","% bias ML 18","% bias ML y19","% bias ML y20",

"% bias ML f12","% bias ML f13","% bias ML f14","% bias ML f23","% bias ML f24","% bias ML f34",

"% bias MLR y1","% bias MLR y2","% bias MLR y3","% bias MLR y4","% bias MLR y5",

"% bias MLR y6","% bias MLR y7","% bias MLR y8","% bias MLR y9","% bias MLR y10",

"% bias MLR y11","% bias MLR y12","% bias MLR y13","% bias MLR y14","% bias MLR y15",

"% bias MLR y16","% bias MLR y17","% bias MLR 18","% bias MLR y19","% bias MLR y20",

"% bias MLR f12","% bias MLR f13","% bias MLR f14","% bias MLR f23","% bias MLR f24","% bias MLR f34",

"% bias ULS y1","% bias ULS y2","% bias ULS y3","% bias ULS y4","% bias ULS y5",

"% bias ULS y6","% bias ULS y7","% bias ULS y8","% bias ULS y9","% bias ULS y10",

"% bias ULS y11","% bias ULS y12","% bias ULS y13","% bias ULS y14","% bias ULS y15",

"% bias ULS y16","% bias ULS y17","% bias ULS 18","% bias ULS y19","% bias ULS y20",

"% bias ULS f12","% bias ULS f13","% bias ULS f14","% bias ULS f23","% bias ULS f24","% bias ULS f34",

"% bias WLS y1","% bias WLS y2","% bias WLS y3","% bias WLS y4","% bias WLS y5",

"% bias WLS y6","% bias WLS y7","% bias WLS y8","% bias WLS y9","% bias WLS y10",

"% bias WLS y11","% bias WLS y12","% bias WLS y13","% bias WLS y14","% bias WLS y15",

"% bias WLS y16","% bias WLS y17","% bias WLS 18","% bias WLS y19","% bias WLS y20",

"% bias WLS f12","% bias WLS f13","% bias WLS f14","% bias WLS f23","% bias WLS f24","% bias WLS f34",

"RMSR WLSpb y1","RMSR WLSpb y2","RMSR WLSpb y3","RMSR WLSpb y4","RMSR WLSpb y5",

"RMSR WLSpb y6","RMSR WLSpb y7","RMSR WLSpb y8","RMSR WLSpb y9","RMSR WLSpb y10",

"RMSR WLSpb y11","RMSR WLSpb y12","RMSR WLSpb y13","RMSR WLSpb y14","RMSR WLSpb y15",

"RMSR WLSpb y16","RMSR WLSpb y17","RMSR WLSpb 18","RMSR WLSpb y19","RMSR WLSpb y20",

"RMSR WLSpb f12","RMSR WLSpb f13","RMSR WLSpb f14","RMSR WLSpb f23","RMSR WLSpb f24","RMSR WLSpb f34",

"RMSR MLpb y1","RMSR MLpb y2","RMSR MLpb y3","RMSR MLpb y4","RMSR MLpb y5",

"RMSR MLpb y6","RMSR MLpb y7","RMSR MLpb y8","RMSR MLpb y9","RMSR MLpb y10",

"RMSR MLpb y11","RMSR MLpb y12","RMSR MLpb y13","RMSR MLpb y14","RMSR MLpb y15",

"RMSR MLpb y16","RMSR MLpb y17","RMSR MLpb 18","RMSR MLpb y19","RMSR MLpb y20",

"RMSR MLpb f12","RMSR MLpb f13","RMSR MLpb f14","RMSR MLpb f23","RMSR MLpb f24","RMSR MLpb f34",

"RMSR ULSpb y1","RMSR ULSpb y2","RMSR ULSpb y3","RMSR ULSpb y4","RMSR ULSpb y5",

"RMSR ULSpb y6","RMSR ULSpb y7","RMSR ULSpb y8","RMSR ULSpb y9","RMSR ULSpb y10",

"RMSR ULSpb y11","RMSR ULSpb y12","RMSR ULSpb y13","RMSR ULSpb y14","RMSR ULSpb y15",

"RMSR ULSpb y16","RMSR ULSpb y17","RMSR ULSpb 18","RMSR ULSpb y19","RMSR ULSpb y20",

"RMSR ULSpb f12","RMSR ULSpb f13","RMSR ULSpb f14","RMSR ULSpb f23","RMSR ULSpb f24","RMSR ULSpb f34",

"RMSR ML y1","RMSR ML y2","RMSR ML y3","RMSR ML y4","RMSR ML y5",

"RMSR ML y6","RMSR ML y7","RMSR ML y8","RMSR ML y9","RMSR ML y10",

"RMSR ML y11","RMSR ML y12","RMSR ML y13","RMSR ML y14","RMSR ML y15",

"RMSR ML y16","RMSR ML y17","RMSR ML 18","RMSR ML y19","RMSR ML y20",

"RMSR ML f12","RMSR ML f13","RMSR ML f14","RMSR ML f23","RMSR ML f24","RMSR ML f34",

"RMSR MLR y1","RMSR MLR y2","RMSR MLR y3","RMSR MLR y4","RMSR MLR y5",

"RMSR MLR y6","RMSR MLR y7","RMSR MLR y8","RMSR MLR y9","RMSR MLR y10",

"RMSR MLR y11","RMSR MLR y12","RMSR MLR y13","RMSR MLR y14","RMSR MLR y15",

"RMSR MLR y16","RMSR MLR y17","RMSR MLR 18","RMSR MLR y19","RMSR MLR y20",

"RMSR MLR f12","RMSR MLR f13","RMSR MLR f14","RMSR MLR f23","RMSR MLR f24","RMSR MLR f34",

"RMSR ULS y1","RMSR ULS y2","RMSR ULS y3","RMSR ULS y4","RMSR ULS y5",

"RMSR ULS y6","RMSR ULS y7","RMSR ULS y8","RMSR ULS y9","RMSR ULS y10",

"RMSR ULS y11","RMSR ULS y12","RMSR ULS y13","RMSR ULS y14","RMSR ULS y15",

"RMSR ULS y16","RMSR ULS y17","RMSR ULS 18","RMSR ULS y19","RMSR ULS y20",

"RMSR ULS f12","RMSR ULS f13","RMSR ULS f14","RMSR ULS f23","RMSR ULS f24","RMSR ULS f34",

"RMSR WLS y1","RMSR WLS y2","RMSR WLS y3","RMSR WLS y4","RMSR WLS y5",

"RMSR WLS y6","RMSR WLS y7","RMSR WLS y8","RMSR WLS y9","RMSR WLS y10",

"RMSR WLS y11","RMSR WLS y12","RMSR WLS y13","RMSR WLS y14","RMSR WLS y15",

"RMSR WLS y16","RMSR WLS y17","RMSR WLS 18","RMSR WLS y19","RMSR WLS y20",

"RMSR WLS f12","RMSR WLS f13","RMSR WLS f14","RMSR WLS f23","RMSR WLS f24","RMSR WLS f34",

"MeanAbs WLSpb y1","MeanAbs WLSpb y2","MeanAbs WLSpb y3","MeanAbs WLSpb y4","MeanAbs WLSpb y5",

"MeanAbs WLSpb y6","MeanAbs WLSpb y7","MeanAbs WLSpb y8","MeanAbs WLSpb y9","MeanAbs WLSpb y10",

"MeanAbs WLSpb y11","MeanAbs WLSpb y12","MeanAbs WLSpb y13","MeanAbs WLSpb y14","MeanAbs WLSpb y15",

"MeanAbs WLSpb y16","MeanAbs WLSpb y17","MeanAbs WLSpb 18","MeanAbs WLSpb y19","MeanAbs WLSpb y20",

"MeanAbs WLSpb f12","MeanAbs WLSpb f13","MeanAbs WLSpb f14","MeanAbs WLSpb f23","MeanAbs WLSpb f24","MeanAbs WLSpb f34",

"MeanAbs MLpb y1","MeanAbs MLpb y2","MeanAbs MLpb y3","MeanAbs MLpb y4","MeanAbs MLpb y5",

"MeanAbs MLpb y6","MeanAbs MLpb y7","MeanAbs MLpb y8","MeanAbs MLpb y9","MeanAbs MLpb y10",

"MeanAbs MLpb y11","MeanAbs MLpb y12","MeanAbs MLpb y13","MeanAbs MLpb y14","MeanAbs MLpb y15",

"MeanAbs MLpb y16","MeanAbs MLpb y17","MeanAbs MLpb 18","MeanAbs MLpb y19","MeanAbs MLpb y20",

"MeanAbs MLpb f12","MeanAbs MLpb f13","MeanAbs MLpb f14","MeanAbs MLpb f23","MeanAbs MLpb f24","MeanAbs MLpb f34",

"MeanAbs ULSpb y1","MeanAbs ULSpb y2","MeanAbs ULSpb y3","MeanAbs ULSpb y4","MeanAbs ULSpb y5",

"MeanAbs ULSpb y6","MeanAbs ULSpb y7","MeanAbs ULSpb y8","MeanAbs ULSpb y9","MeanAbs ULSpb y10",

"MeanAbs ULSpb y11","MeanAbs ULSpb y12","MeanAbs ULSpb y13","MeanAbs ULSpb y14","MeanAbs ULSpb y15",

"MeanAbs ULSpb y16","MeanAbs ULSpb y17","MeanAbs ULSpb 18","MeanAbs ULSpb y19","MeanAbs ULSpb y20",

"MeanAbs ULSpb f12","MeanAbs ULSpb f13","MeanAbs ULSpb f14","MeanAbs ULSpb f23","MeanAbs ULSpb f24","MeanAbs ULSpb f34",

"MeanAbs ML y1","MeanAbs ML y2","MeanAbs ML y3","MeanAbs ML y4","MeanAbs ML y5",

"MeanAbs ML y6","MeanAbs ML y7","MeanAbs ML y8","MeanAbs ML y9","MeanAbs ML y10",

"MeanAbs ML y11","MeanAbs ML y12","MeanAbs ML y13","MeanAbs ML y14","MeanAbs ML y15",

"MeanAbs ML y16","MeanAbs ML y17","MeanAbs ML 18","MeanAbs ML y19","MeanAbs ML y20",

"MeanAbs ML f12","MeanAbs ML f13","MeanAbs ML f14","MeanAbs ML f23","MeanAbs ML f24","MeanAbs ML f34",

"MeanAbs MLR y1","MeanAbs MLR y2","MeanAbs MLR y3","MeanAbs MLR y4","MeanAbs MLR y5",

"MeanAbs MLR y6","MeanAbs MLR y7","MeanAbs MLR y8","MeanAbs MLR y9","MeanAbs MLR y10",

"MeanAbs MLR y11","MeanAbs MLR y12","MeanAbs MLR y13","MeanAbs MLR y14","MeanAbs MLR y15",

"MeanAbs MLR y16","MeanAbs MLR y17","MeanAbs MLR 18","MeanAbs MLR y19","MeanAbs MLR y20",

"MeanAbs MLR f12","MeanAbs MLR f13","MeanAbs MLR f14","MeanAbs MLR f23","MeanAbs MLR f24","MeanAbs MLR f34",

"MeanAbs ULS y1","MeanAbs ULS y2","MeanAbs ULS y3","MeanAbs ULS y4","MeanAbs ULS y5",

"MeanAbs ULS y6","MeanAbs ULS y7","MeanAbs ULS y8","MeanAbs ULS y9","MeanAbs ULS y10",

"MeanAbs ULS y11","MeanAbs ULS y12","MeanAbs ULS y13","MeanAbs ULS y14","MeanAbs ULS y15",

"MeanAbs ULS y16","MeanAbs ULS y17","MeanAbs ULS 18","MeanAbs ULS y19","MeanAbs ULS y20",

"MeanAbs ULS f12","MeanAbs ULS f13","MeanAbs ULS f14","MeanAbs ULS f23","MeanAbs ULS f24","MeanAbs ULS f34",

"MeanAbs WLS y1","MeanAbs WLS y2","MeanAbs WLS y3","MeanAbs WLS y4","MeanAbs WLS y5",

"MeanAbs WLS y6","MeanAbs WLS y7","MeanAbs WLS y8","MeanAbs WLS y9","MeanAbs WLS y10",

"MeanAbs WLS y11","MeanAbs WLS y12","MeanAbs WLS y13","MeanAbs WLS y14","MeanAbs WLS y15",

"MeanAbs WLS y16","MeanAbs WLS y17","MeanAbs WLS 18","MeanAbs WLS y19","MeanAbs WLS y20",

"MeanAbs WLS f12","MeanAbs WLS f13","MeanAbs WLS f14","MeanAbs WLS f23","MeanAbs WLS f24","MeanAbs WLS f34"

)

colnames(bias.rep) <- col5

rownames(fit.rep) <- cond.row

rownames(dec.rep) <- cond.row

rownames(para.rep) <- cond.row

rownames(bias.rep) <- cond.row

write.table(fit.rep, paste(dir,"fit.csv",sep=""),col.names=NA,sep=",")

write.table(dec.rep, paste(dir,"decision.csv",sep=""),col.names=NA,sep=",")

write.table(para.rep, paste(dir,"parameters.csv",sep=""),col.names=NA,sep=",")

write.table(parase.rep, paste(dir,"parametersSE.csv",sep=""),col.names=NA,sep=",")

write.table(bias.rep, paste(dir,"bias.csv",sep=""),col.names=NA,sep=",")

write.table(cond.save, paste(dir,"conditions.csv",sep=""),col.names=NA,sep=",")

write.table(truepara.rep, paste(dir,"trueparameters.csv",sep=""),row.names=FALSE,col.names=FALSE,sep=",")

write.table(restart.rep, paste(dir,"restart.csv",sep=""),col.names=NA,sep=",")
