# Supplementary material for: Probability-of-Superiority SEM (PS-SEM)—Detecting Probability-Based Multivariate Relationships in Behavioral Research
Source: Front Psychol. 2018 Jun 13;9:883. doi: 10.3389/fpsyg.2018.00883 (PMC6008517; doi:10.3389/fpsyg.2018.00883)
Supplement: Supplementary file 2 [file Data_Sheet_2.docx]

**Appendix A – Working Example**

**Step 1. Download the dataset** (file name is “data.csv”) from http://openpsychometrics.org/_rawdata/AS+SC+AD+DO.zip, open “data.csv”, select only the variables that are used in the analysis (i.e., AS7, AS8, SC1, SC4, DO4, DO5, AD7, AD6, age, and gender), and save it as “datasel4.csv”. Import “datasel4.csv” to R or RStudio.

**Step 2. Execute the following function that can perform a PS-SEM analysis**

library(lavaan)

library(psych)

require(MASS)

require(lavaan)

require(GenOrd)

library(distr)

library(truncdist)

library(GenOrd)

library(corpcor)

options(scipen=999)

data <- fdata

#pr is the function that generates the PS-adjusted correlation or covariance matrix as input in SEM

pr<-function(data, cor = FALSE){

p <- dim(data)[2]

n <<-length(data[,1])

re <- array(1,dim=c(p,p))

for(i in 1:p){

tem=i+1

if(tem<=p){

for(j in tem:p){

diff<-(data[,i]-mean(data[,i]))*(data[,j]-mean(data[,j]))

tcount<- length(as.integer(which(diff>0)))+length(as.integer(which(diff==0)))/2

re[i,j] <- tcount/n

}}}

ator <- function(a){return(sin(pi*(a-0.5)))}

f <- function(m) {

m[lower.tri(m)] <- t(m)[lower.tri(m)]

m

}

re <- ator(re)

re <- f(re)

if(cor==FALSE){

for(i in 1:p){

re[i,i] <- var(data[,i])

tem=i+1

if(tem<=p){

for(j in tem:p){

re[i,j] <- re[i,j]*sd(data[,i])*sd(data[,j])

}}}

re <- f(re)}

label <- colnames(data)

mu <<- rep(0,p)

check4 <- is.positive.definite(re)

if(check4 == TRUE){

wdat <- mvrnorm(20000,mu,re)

colnames(wdat) <- label

tfit <- cfa(HS.model, data=wdat, estimator = "WLS")

weightmatrix <<- inspect(tfit, "WLS.V")}

colnames(re) <- label

rownames(re) <- label

return(data.matrix(re))}

#Real-World Example

data <- data.frame(datasel4)

#Delete Missing Values or Unused Categories in Some Variables

data$gender[data$gender=="3"]<-0 #There are 2 cases with “3 = other” in gender

data[data=="0"]<-NA #0 is the category reserved for storing a missing value in the original data

data <- data[!is.na(data$AS7), ]

data <- data[!is.na(data$AS8), ]

data <- data[!is.na(data$SC1), ]

data <- data[!is.na(data$SC4), ]

data <- data[!is.na(data$DO4), ]

data <- data[!is.na(data$DO5), ]

data <- data[!is.na(data$AD6), ]

data <- data[!is.na(data$AD7), ]

data <- data[!is.na(data$age), ]

data <- data[!is.na(data$gender), ]

#Specify the Model

HS.model <<- 'AS =~ AS7 + AS8

SC =~ SC1 + SC4

DO =~ DO4 + DO5

AD =~ AD6 + AD7

AS ~ age+gender

SC ~ age+gender

DO~ age+gender

AD ~ age+gender'

data <- data.frame(data)

fit1 <- cfa(HS.model, estimator="ML", sample.cov = pr(data), sample.nobs = n, std.lv=TRUE) #PS-ML

summary(fit1, standardized = TRUE, fit.measures= TRUE)

**Step 3 Obtain the Results**

> fit1 <- cfa(HS.model, estimator="ML", sample.cov = pr(data), sample.nobs = n, std.lv=TRUE)

> summary(fit1, standardized = TRUE, fit.measures= TRUE)

lavaan (0.5-23.1097) converged normally after 46 iterations

Number of observations 973

Estimator ML

Minimum Function Test Statistic 165.216

Degrees of freedom 22

P-value (Chi-square) 0.000

Model test baseline model:

Minimum Function Test Statistic 3304.090

Degrees of freedom 44

P-value 0.000

User model versus baseline model:

Comparative Fit Index (CFI) 0.956

Tucker-Lewis Index (TLI) 0.912

Loglikelihood and Information Criteria:

Loglikelihood user model (H0) -14040.332

Loglikelihood unrestricted model (H1) -13957.724

Number of free parameters 30

Akaike (AIC) 28140.665

Bayesian (BIC) 28287.076

Sample-size adjusted Bayesian (BIC) 28191.796

Root Mean Square Error of Approximation:

RMSEA 0.082

90 Percent Confidence Interval 0.070 0.094

P-value RMSEA <= 0.05 0.000

Standardized Root Mean Square Residual:

SRMR 0.034

Parameter Estimates:

Information Expected

Standard Errors Standard

Latent Variables:

Estimate Std.Err z-value P(>|z|) Std.lv Std.all

AS =~

AS7 0.818 0.038 21.770 0.000 0.855 0.870

AS8 0.632 0.033 19.304 0.000 0.660 0.693

SC =~

SC1 0.870 0.040 22.000 0.000 0.876 0.808

SC4 0.761 0.036 20.938 0.000 0.766 0.756

DO =~

DO4 0.810 0.047 17.335 0.000 0.827 0.786

DO5 0.820 0.048 17.093 0.000 0.838 0.758

AD =~

AD6 0.921 0.029 31.571 0.000 0.933 0.924

AD7 0.878 0.027 32.076 0.000 0.889 0.938

Regressions:

Estimate Std.Err z-value P(>|z|) Std.lv Std.all

AS ~

age -0.026 0.003 -7.611 0.000 -0.024 -0.273

gender 0.235 0.073 3.207 0.001 0.224 0.112

SC ~

age 0.009 0.003 2.830 0.005 0.009 0.104

gender 0.091 0.074 1.234 0.217 0.091 0.045

DO ~

age -0.012 0.003 -3.535 0.000 -0.012 -0.130

gender -0.316 0.076 -4.180 0.000 -0.309 -0.154

AD ~

age 0.008 0.003 2.597 0.009 0.008 0.086

gender 0.261 0.067 3.889 0.000 0.258 0.129

Covariances:

Estimate Std.Err z-value P(>|z|) Std.lv Std.all

.AS ~~

.SC -0.354 0.038 -9.208 0.000 -0.354 -0.354

.DO -0.359 0.039 -9.216 0.000 -0.359 -0.359

.AD 0.241 0.036 6.684 0.000 0.241 0.241

.SC ~~

.DO 0.137 0.042 3.235 0.001 0.137 0.137

.AD -0.471 0.032 -14.945 0.000 -0.471 -0.471

.DO ~~

.AD 0.004 0.039 0.111 0.912 0.004 0.004

Variances:

Estimate Std.Err z-value P(>|z|) Std.lv Std.all

.AS7 0.235 0.050 4.692 0.000 0.235 0.243

.AS8 0.472 0.036 13.035 0.000 0.472 0.519

.SC1 0.409 0.052 7.884 0.000 0.409 0.347

.SC4 0.439 0.042 10.462 0.000 0.439 0.428

.DO4 0.424 0.066 6.409 0.000 0.424 0.382

.DO5 0.521 0.069 7.550 0.000 0.521 0.426

.AD6 0.149 0.031 4.732 0.000 0.149 0.146

.AD7 0.109 0.028 3.831 0.000 0.109 0.121

.AS 1.000 0.915 0.915

.SC 1.000 0.987 0.987

.DO 1.000 0.958 0.958

.AD 1.000 0.975 0.975

**Step 4. Convert the Estimates to PS Metric**

rtoa <- function(r){return(asin(r)/pi+0.5)}

loadings <- c(.87, .693, .808, .756, .786, .758, .924, .938)

rtoa(loadings)

reg <- c(-0.273, .112, .104, .045, -.13, -.154, .086, .129)

rtoa(reg)

corr <- c(-.354, -.359, .241, .137, -.471, .004)

rtoa(corr)

> rtoa(loadings)

[1] 0.8358813 0.7437114 0.7994499 0.7728490 0.7878514 0.7738233

[7] 0.8751002 0.8873242

> rtoa(reg)

[1] 0.4119841 0.5357257 0.5331642 0.5143288 0.4585023 0.4507844

[7] 0.5274085 0.5411767

> rtoa(corr)

[1] 0.3848213 0.3831178 0.5774754 0.5437460 0.3438932 0.5012732
